# Identification of Antibody and Small Molecule Antagonists of Ferroportin-Hepcidin Interaction

**DOI:** 10.3389/fphar.2017.00838

**Published:** 2017-11-21

**Authors:** Sandra L. Ross, Kaustav Biswas, James Rottman, Jennifer R. Allen, Jason Long, Les P. Miranda, Aaron Winters, Tara L. Arvedson

**Affiliations:** ^1^Department of Oncology Research, Amgen Inc., Thousand Oaks, CA, United States; ^2^Department of Hybrid Modality Engineering, Amgen Inc., Thousand Oaks, CA, United States; ^3^Department of Comparative Biology and Safety Sciences, Amgen Inc., Thousand Oaks, CA, United States; ^4^Department of Medicinal Chemistry, Amgen Inc., Thousand Oaks, CA, United States; ^5^Department of Therapeutic Discovery, Amgen Inc., Thousand Oaks, CA, United States

**Keywords:** ferroportin, hepcidin, iron metabolism, receptor internalization, antibody engineering, anti-ferroportin monoclonal antibody, fluorescently-labeled hepcidin, ferroportin antagonist

## Abstract

The iron exporter ferroportin and its ligand, the hormone hepcidin, control fluxes of stored and recycled iron for use in a variety of essential biochemical processes. Inflammatory disorders and malignancies are often associated with high hepcidin levels, leading to ferroportin down-regulation, iron sequestration in tissue macrophages and subsequent anemia. The objective of this research was to develop reagents to characterize the expression of ferroportin, the interaction between ferroportin and hepcidin, as well as to identify novel ferroportin antagonists capable of maintaining iron export in the presence of hepcidin. Development of investigative tools that enabled cell-based screening assays is described in detail, including specific and sensitive monoclonal antibodies that detect endogenously-expressed human and mouse ferroportin and fluorescently-labeled chemically-synthesized human hepcidin. Large and small molecule antagonists inhibiting hepcidin-mediated ferroportin internalization were identified, and unique insights into the requirements for interaction between these two key iron homeostasis molecules are provided.

## Introduction

Ferroportin (FPN, ferroportin 1, also known as IREG1, MTP1, SLC40A1), the only recognized mammalian iron exporter protein, is required for dietary iron uptake and mobilization of iron from tissues. Iron readily cycles between oxidation states at physiological pH, and as a result it has essential roles in electron transfer and oxygen consumption, DNA replication and repair, ribosome maturation and cell cycle progression (Zhang, 2014). However, this redox activity also has the potential to generate reactive oxygen species, and therefore iron movement must be tightly controlled to prevent toxic side effects (Andrews, 1999; Zhang, 2014). Hepcidin (HEPC, also known as LEAP1) is synthesized in the liver in response to inflammation and increased tissue iron (Nicolas et al., 2002) as an 84-amino acid prepropeptide that is processed to 60-amino acid prohepcidin; cleavage of the signal peptide produces a 25-amino acid peptide (Park et al., 2001). Hepcidin is the only recognized ligand for FPN and regulates FPN activity by inducing its internalization and proteolysis (Nemeth et al., 2004).

FPN is highly expressed on the basolateral membrane of duodenal enterocytes, where it transports dietary iron to the bloodstream, on the plasma membrane of macrophages where it mediates release of iron recycled from senescent erythrocytes and on the sinusoidal surface of hepatocytes, where it transports iron stored in the liver to the plasma (Donovan et al., 2000; Canonne-Hergaux et al., 2006; Ramey et al., 2010). Chronic elevated levels of serum hepcidin result in sustained down-regulation of enterocyte and macrophage FPN, leading to reduction in dietary iron absorption and iron sequestration in macrophages, where it is inaccessible for hemoglobinization of new red blood cells. Thus, patients can be iron replete and still be anemic. FPN may be a suitable target for treating chronic inflammatory anemia if a therapeutic means of preventing hepcidin-mediated internalization can be identified. Agents neutralizing hepcidin have been described and are being evaluated clinically; however, it could be challenging to effectively neutralize a soluble ligand that is present at high concentration in disease settings. Human blood and urine hepcidin assays are still evolving and include ELISA, RIA and mass spectrometry. Measurement of absolute hepcidin concentrations is highly variable due to lack of standardization across assay platforms (Macdougall et al., 2010; Arezes and Nemeth, 2015). For example, the mean serum hepcidin levels measured in different assays for chronic kidney disease (CKD) patients on hemodialysis range from 9 to 242 nM (Macdougall et al., 2010). Given this uncertainty, targeting FPN may offer a therapeutic advantage. Agents reported to inhibit hepcidin-mediated degradation of FPN have been described, including anti-FPN antibodies (Leung et al., 2014) and small molecules (Fung et al., 2013); however none of these agents have met efficacy criteria as single agents in ongoing studies in humans.

To enable FPN characterization and support assay development, we developed reagents that bind to surface-expressed FPN, including Rhodamine G-labeled hepcidin (RhoG-hepc) and monoclonal antibodies against both human and mouse FPN. Using assays that incorporate these reagents, antibody and small molecule panels were screened and agents that protect against hepcidin-induced FPN internalization were identified. Herein we describe these novel reagents and how they were used to extend the understanding of the FPN-hepcidin interaction.

## Materials and methods

### Cell lines

T-REx™/FPN-V5 cells were generated as described (Ross et al., 2012). T-REx™/FPN-V5 cells were engineered with a ß-lactamase (BLA) reporter gene joined to the 5′UTR [containing one copy of the iron response element (IRE)] of ferritin to generate the T-REx™/FPN-V5/IRE-BLA cell line. The nucleotide sequence of the ferritin IRE is provided in the international patent application WO 2009/094551 A1 (Arvedson et al., 2009). IRE-BLA sequences were cloned into pENTR1A and stably transfected into T-REx™/FPN-V5 cells. 293 T-REx™/FPN-V5, T47D, and UT-7/Epo cell lines were cultured as described (Ross et al., 2012).

### Commercial antibodies

Rabbit anti-Cyclophilin B antibody (Abcam, #ab16045) was used at 0.5 μg/ml as a loading control for immunoblots. Rabbit anti-V5 antibody (Abcam #9116) was used at 1 μg/ml as a control for FPN from engineered cell lines in immunoblots. Tested anti-FPN antibodies include Alpha Diagnostics (MTP11-A, used at 5 μg/ml) and LifeSpan Bioscience (LS-B1836S, used at 1.5 μg/ml) for immunoblotting. Anti-CD68 (Santa Cruz #sc-9139), anti-GFAP (Biocare #040) rabbit polyclonal antibodies were used for staining of macrophages and astrocytes, respectively, followed by goat-anti-rabbit Alexa-fluor® 594 (Invitrogen/Thermo Fisher #A-11012).

### Ferroportin antibody generation

Mouse anti-human FPN antibodies were generated in C57BL/6 mice (Charles River Laboratories) by immunizing with either membranes from human FPN-expressing Chinese Hamster Ovary (CHO) cells or with recombinant adenoviral vector carrying human FPN. Mouse anti-human FPN antibody 31A5 was generated by immunizing C57BL/6 mice (Charles River Laboratory) with an adenovirus expressing human FPN and boosted with FPN DNA as described in the published international patent application WO 2009/094551 A1 (Arvedson et al., 2009). Fully human antibodies were generated in XenoMouse™ (Mendez et al., 1997) by immunizing mice with membranes from HEK293-6E cells expressing FPN using pTT5 plasmid (cells and plasmid licensed from the National Research Council of Canada Biotechnology Research Institute). Hybridomas were generated using standard techniques and clones expressing antibodies that bound the extracellular region of FPN were selected by screening hybridoma supernatants against live HEK 293T cells expressing inducible human FPN (T-REx™/Fpn-V5). Anti-mouse FPN antibodies were generated by immunizing rats with membranes from mouse FPN-expressing CHO cells. Mouse FPN pTT5 constructs were designed with and without PADRE T cell epitope tags and the protein transiently expressed in HEK293 cells. Expression cell pools were harvested and further processed into membrane preparations for use as antigen. Groups of Brown Norway rats (Charles River) were immunized 3–5 times with 100–200 μg per immunization of mouse FPN-PADRE membranes emulsified in the following adjuvants: complete Freund's adjuvant (CFA, Sigma), incomplete Freund's adjuvant (IFA, Sigma), Alum (Thermo Fisher), CpG (Amgen). Serum titers were analyzed by flow cytometry (BD) on CHO cells expressing mouse FPN and mock-transfected CHO cells. Spleens from rats that showed the best serum titer response were harvested for hybridoma generation by electrofusion. Due to contaminating host cell immune responses arising from immunizing with mouse FPN membrane preparations, bacolovirus mouse FPN-V5 constructs were expressed in TNi insect cells. Mouse FPN-expressing TNi cells in conjunction with goat anti-rat IgG, Fc specific-647 secondary antibodies (Jackson ImmunoResearch) were used to screen hybridoma supernatants, immune rat serum, and purified IgGs on the FMAT platform (Applied Biosystems) as well as by flow cytometry (BD).

### Epitope mapping

Peptides of 10 amino acids were synthesized using a MultiPep instrument (Intavis) directly on cellulose membranes (Intavis) using the peptide spot (PepSpot) array technique (Briant et al., 2009). Antibodies were screened against individual overlapping FPN peptides (10 amino acids in length over the entire length of the FPN sequence) immobilized on membranes in individual spots. The first spot contained a peptide with amino acids 1–10, the second spot contained a peptide with amino acids 2–11, followed by 3–12, and so on. This pattern was followed over the length of the ferroportin sequence to ensure the entire protein was represented. Membranes were soaked in methanol for 5 min, washed twice in Tris-buffered saline, 0.1% Tween 20 (TBST) buffer, and blocked with 5% non-fat dried milk (NFDM) in TBST. Antibodies were screened at 1 μg/ml in 5% NFDM for 1 h with shaking at room temperature. After washing four times in TBST, membranes were incubated with goat anti-human IgG-HRP (Pierce/Thermo Fisher) secondary antibody at 1:50,000 in 5% NFDM. After washing four times in TBST, spots were detected by enhanced chemiluminescence (ECL, Pierce/Thermo Fisher).

### Immunofluorescence and cellular imaging assays

Cells were plated in 96-well Poly-D-Lysine coated plates (BD) at 50,000 cells/well and induced overnight with 10 ng/ml doxycycline (Clontech). Cell surface FPN and RhoG-hepcidin uptake assays were performed as described (Ross et al., 2012). Briefly, induction medium was replaced with assay medium (DMEM/10% dialyzed FBS (for T-REx™/FPN-V5 cells) or growth medium (for all other cell lines) containing hepcidin. Cells were incubated at 37°C at varying concentrations of hepcidin for varying times (generally 30–360 min). For cell surface FPN detection, hepcidin-containing medium was replaced with anti-FPN antibodies at concentrations listed in figure legends in cold assay medium, incubated for 1 h at 4°C, washed twice, and incubated for 1 h with secondary antibodies (goat anti-mouse-Alexa Fluor 647 or goat anti-human-Alexa Fluor 647, Invitrogen/Thermo Fisher) and Hoechst 33342 nuclear dye (Invitrogen/Thermo Fisher). After staining, cells were fixed with 3.7% formaldehyde solution (Sigma) for 10 min at room temperature and washed with phosphate-buffered saline (PBS, GIBCO). For total FPN staining, cells were fixed with 3.7% formaldehyde solution and permeabilized with 0.1% saponin (Calbiochem) and 1% bovine serum albumin (Sigma) in PBS at room temperature. FPN staining was completed as described above except that the incubations were performed at room temperature. For RhoG-hepcidin uptake assays, T-REx™/FPN-V5 cells were treated with 250 nM RhoG-Hepc for varying times (generally 30–360 min.) and fixed with 3.7% formaldehyde. Nuclei were stained with Hoechst dye. Cell surface or total FPN or intracellular hepcidin was quantitated with cellular imaging. Uninduced (non-FPN-expressing) cells served as a negative control. Images were captured from Arrayscan™ VTI (Thermo Fisher) equipped with Zeiss microscope optics and Hamamatsu ORCA®-ER CCD camera. Images were captured with 10X or 20X objectives and analyzed with the Spot Detector bioapplication. A minimum of 300 cells per well were analyzed for each condition.

### Immunoblotting

Cells were lysed in 20 mM Tris (pH 7.5), 150 mM NaCl, 1 mM EDTA, 1 mM EGTA, 1% Triton X-100 with protease and phosphatase inhibitors (NaF, Roche protease inhibitor, and Sigma phosphatase inhibitors I and II). Protein was quantitated using a BCA protein assay (Thermo Fisher). For FPN analysis, non-reduced lysates were heated at 37°C for 10 min prior to loading on a 4–20% Tris-Glycine gel (Novex) and transferred to nitrocellulose membranes. FPN was detected with anti-FPN antibodies at concentrations listed in Figure Legends. Human cyclophilin B (CypB, rabbit polyclonal antibody, Abcam) was used as a protein loading control. HRP-labeled, species-specific secondary antibodies were used for detection (Cell Signaling).

### siRNA knockdown

T-REx™/FPN-V5 cells were reverse transfected with 20 nM SLC40A1 (Invitrogen/Thermo Fisher #HSS121213), cyclophilin B (QIAGEN, custom), or All Stars negative control (QIAGEN #1027281) siRNAs using RNAiMAX transfection reagent (Invitrogen/Thermo Fisher) according to the manufacturer's protocol. Cells were induced to express FPN with 10 ng/ml doxycycline immediately before transfection. Lysates were made 48 h after transfection. Protein (20 mg) from each sample was tested by Western analysis as described.

### Human tissues

All human specimens were collected under Institutional Review Board approval with appropriate informed consent. In all cases, materials obtained were surplus to standard clinical practice. Patient identity and PHI/identifying information were redacted from tissues and clinical data. Human tissue specimens were obtained from the following institutions: Asterand Bioscience, Detroit, MI and Zoion, Hawthorne, NY.

### Ferroportin immunohistochemistry

Formalin-fixed, paraffin-embedded sections of human tissue were stained with a mouse monoclonal anti-human FPN Ab 31A5. Tissue sections were stained via indirect immunofluorescence or with the ABC-peroxidase technique. Brightfield mages were collected with a Nikon Ni-U microscope and a DS-Fi2 camera. Fluorescent images were collected with a Nikon Eclipse 50i fluorescence microscope illuminated by an EXFO Excite 120 light source and acquired using NIS elements v3.0 software.

### *In situ* hybridization

Human FPN probe: A 389 bp fragment of the human FPN gene, corresponding to nucleotides 1632–2020 (Genbank #AF226614.1), was cloned into the pCR4-TOPO plasmid vector (Thermo Fisher). The identity of the template was verified by sequencing. An antisense ^33^P-labeled RNA probe was synthesized by *in vitro* transcription of the template with T3 RNA polymerase after linearization of the vector with Not I restriction enzyme. A ^33^P-labeled sense probe was also generated from the same template using T7 RNA polymerase and Spe I restriction enzyme. All of the tissue used in the study was derived from archived blocks of immersion fixed, paraffin embedded material from which 5 μm sections were taken. A standard ISH protocol (Wilcox, 1993) was performed involving overnight hybridization at 60°C in a hybridization solution containing 1 × 10^6^ cpm of ^33^P-labeled riboprobe per slide. To improve target detection, all tissue slides were subjected to a pretreatment by microwave heating to 100°C totaling 10 min in a citric acid buffer solution (Citra—Biogenex) prior to hybridization. After overnight hybridization all slides were subjected to RNase digestion followed by a series of SSC washes with the highest stringency of 0.1X SSC at 55°C for 30 min. The slides were coated with Kodak NTB emulsion and exposed for 3 weeks in the dark at 4°C, developed, and then counterstained with hematoxylin and eosin.

### Knock-in mice

Human FPN cDNA was targeted at the ATG starting codon of the mouse FPN locus, and ended at the stop codon, keeping all of the 3′UTR of the mouse gene intact, and replacing the entire mouse FPN locus with human FPN cDNA. The FPN cDNA with Neo selection cassette inserted at the 3′ end of the FPN gene was flanked by homology arms. The floxed Neo cassette was removed by *cre* recombinase in 129Sv (agouti) embryonic stem (ES) cells. ES cell clones were karyotyped and microinjected into C57BL/6 blastocyst embryos. Chimeric (129Sv/C57BL/6) blastocysts were microinjected into C57BL/6 mice. Male 8-week old mature chimera (F0) were crossed with female C57BL/6 mice to obtain germline transmitted F1 heterozygotes. Only heterozygous mice were obtained.

### Screening assays

#### β-lactamase assay (BLA) screening assay

T-REx™/FPN-V5/IRE-BLA cells were plated in 384-well Poly-D-Lysine coated plates (BD) at 25,000 cells per well in assay medium (growth medium without selection antibiotics + 2.5 μg/ml ferric citrate) and treated overnight with 10 ng/ml doxycycline to induce FPN expression. Cells were treated with compounds for 1 h prior to adding 36 nM hepcidin followed by overnight incubation. Beta-lactamase activity was detected with fluorescent CCF2 substrate for ß-lactamase (GeneBLAzer®, Thermo Fisher). β-lactamase substrate was added for 4 h. Plates were exposed to 409 nm and emissions read at 447 and 520 nm on an EnVision plate reader (PerkinElmer). Blue/green FRET signal ratio was calculated.

#### RhoG-hepcidin uptake assay

T-REx™/FPN-V5 cells were plated in 384-well Poly-D-Lysine coated plates (BD) at 15,000 cells/well and induced overnight as described for the BLA screening assay. Cells were treated with compound for 1 h prior to adding 250 nM RhoG-hepcidin for 1 h. Plates were washed and fixed with 4% formaldehyde (Thermo Fisher) and nuclei stained with 1 μg/ml Hoechst nuclear dye (Thermo Fisher). Plates were scanned on Thermo Fisher ArrayScan™ HCS Reader and analyzed with Spot Detector application. A minimum of 300 cells/well were analyzed.

#### Ferroportin internalization assay

T-REx™/FPN-V5 were plated in 384-well Poly-D-Lysine coated plates (BD) at 15,000 cell/well and induced overnight as described for RhoG-hepcidin uptake assay. Cells were treated with compound for 1 h prior to adding 250 nM hepcidin for 1 h. Cells were fixed with 4% methanol-free formaldehyde (Thermo Fisher) and stained with 4 μg/ml antibody 38G6-Alexa 647 and 2 μg/ml Hoechst nuclear dye (Thermo Fisher). Plates were scanned on Thermo Fisher ArrayScan™ HCS Reader and analyzed with Spot Detector application. A minimum of 300 cells/well were analyzed.

### RhoG-hepcidin reversibility assay

T-REx™/FPN-V5 cells plated in 96-well Packard ViewPlates at 50,000 cells/well and induced overnight as described for the BLA screening assay. Cells were incubated with compound for 30 min, followed by two washes to remove compound before addition of 250 nM RhoG-hepcidin for 1 h. As a control, cells were treated identically except that compound was not removed before addition of RhoG-hepcidin. Cells were fixed with 4% formaldehyde (Thermo Fisher) and stained with Hoechst nuclear dye. RhoG-Hepc uptake per cell was measured by cellular imaging.

### ^3^H-quinoxaline binding

Induced and uninduced cells were treated ± ^3^H-quinoxaline at 0.33 μM, and incubated at 37°C for 30 min. Unbound quinoxaline was removed by centrifugation. Non-reduced clarified lysates were run on a 4–12% Bis-Tris gel. The gel was cut into 2 parts; one side was dried on a BioRad gel dryer and exposed to autoradiography film for 1 month. The other side was transferred to nitrocellulose membrane and FPN detected with antibody 38C8.

### Glycosylation analysis

T-REx™/FPN-V5 cells were treated ± 0.25–1 μg/ml tunicamycin (Sigma-Aldrich) for 18 h, followed by treatment with 500 nM hepcidin for 5 h for blots or 3 h for a RhoG-hepcidin uptake assay.

### Synthetic human hepcidin and rhodamine green labeled hepcidin (RhoG-Hepc) synthesis

#### Synthesis

Synthetic human hepcidin mutant was synthesized on a CSBio CS336x (CSBio Menlo Park, CA) using *N*^α^-Fmoc/^t^Bu side chain protection with *N,N*'-diisopropylcarbodiimide (DIC)/ 1-hydroxybenzotriazole (HOBT) chemistry in dimethylformamide (DMF) with from deprotection employing 20% (v/v) piperdine in DMF. H-Thr(^t^Bu)-ChemMatrix resin ChemMatrix Quebec, Canada) was used at 0.12 mmol equivalent scale. The following Fmoc protected amino acids were used, Thr(^t^Bu), His(Boc), Phe, Pro, Ile, Cys(Trt), Gly, Arg(Pbf), Lys(ivDde), Lys(Boc), Met, with the exception of Boc-Asp(^t^Bu) at the N-terminus. 5 equivalents of activated amino acid were coupled to deprotected amine for 2 h followed by 2 × 15 min Fmoc deprotection with 20% piperdine. An aminohexanoic (Ahx) spacer was attached to the ε-amine of lysine with 5 equivalents Fmoc-Ahx-OH, (2-(6-Chloro-1H-benzotriazole-1-yl)-1,1,3,3-tetramethylaminium hexafluorophosphate) (HCTU) and DIEA (1:1:1.2) in DMF. Fmoc was removed with 2 × 10 min treatments of 20% piperdine. Rhodamine Green carboxylic acid succinimidyl ester hydrochloride (RhoG, Invitrogen Eugene, OR) was solubilized in DMF with DIEA, 1.5 mmol and 2.0 mmol equivalents, respectively and coupled overnight. The resin was washed with DMF, DCM, and finally ethyl ether.

#### Side chain deprotection/cleavage

Side chain deprotection and resin cleavage was accomplished with a trifluoracetic acid (TFA) solution containing 2.5% water, 2.5% triisopropylsilane (TIS), and 2.5% 3,6-dioxa-1,8-dithiol (DODT) in for 90 min with stirring. The peptide solution was 0.2 um filtered into a 50 mL conical Falcon tube and concentrated *in vacuo*. The peptide was precipitated with cold ethyl ether and centrifuged. The etherate was decanted and the peptide washed twice with ethyl ether and dried in vacuo. Yield = 180 mg (45%)

#### Folding

RhoG-Hepcidin was dissolved in a buffer of 0.18 mM oxidized glutathione (GSSG)/reduced glutathione (GSH) in 30% (v/v) acetonitrile (ACN)/water to a concentration final peptide concentration of 0.03 mM. The pH was adjusted to 8.5 with the addition of 1.0 M Tris(hydroxylmethyl) aminomethane hydrochloride (Tris-HCl) pH 8.5 and stirred overnight ~18 h. The folding was monitored by LCMS. When folding was determined to be complete the solution was quenched by the addition of TFA to pH 2.7.

#### Purification

The quenched folded peptide solution was filtered through a Corning 0.45 μm filter system and the acetonitrile evaporated *in vacuo*. The solution was loaded onto a Synergi MAX-RP 4u 100Ǻ 250 × 30 mm column by loading pump. An elution gradient of 10–40% buffer B (0.1% TFA in ACN) in 45 min at 40 mL^−1^. The fractions were analyzed by liquid chromatography-mass spectrometry (LCMS), fractions containing >95% pure hepcidin were pooled and lyophilized. Calculated mass: 3,299.94 Da, observed mass: 3,299.35 Da. Yield = 30.2 mg (17%).

### Alanine scanning

Synthetic human hepcidin alanine mutants were synthesized on a Tetras asynchronous automated synthesizer (Creosalus Louisville, KY) using *N*^α^-Fmoc/^t^Bu side chain protection with (1-Cyano-2-ethoxy-2-oxoethylidenaminooxy)dimethylamino-morpholino-carbenium hexafluorophosphate (COMU)/ N,N-Diisopropylethylamine (DIEA) (1:1.2) chemistry in dimethylformamide (DMF) with Fmoc deprotection employing 20% (v/v) piperdine in DMF. H-Thr(^t^Bu)-ChemMatrix resin ChemMatrix Quebec, Canada) was used at 0.10 mmol equivalent scale. The following Fmoc protected amino acids were used, Thr(^t^Bu), His(Boc), Phe, Pro, Ile, Cys(Trt), Gly, Arg(Pbf), Lys(ivDde), Lys(Boc), Met, and Asp(^t^Bu). 5 equivalents of activated amino acid were coupled to the deprotected amine for 45 min followed by 2 × 15 min Fmoc deprotection with 20% piperdine in DMF. Alanine mutant peptides were tested for activity in both BLA and ferroportin internalization assays.

### Small molecule reagents

Sulfonyl quinoxaline 1 is 2-[(4-chlorophenyl)sulfonyl]quinoxaline and is commercially available from Key Organics Ltd., Cornwall, UK (CAS #: 338394-53-9; www.keyorganics.net). Tritiated quinoxaline 2 was obtained from Moravek Inc., Brea, CA.

### Mass spectrometry

Peptide and small molecules (100 μM each) were incubated together in 20 mM acetate buffer, pH 7 for 1 h (iodoacetamide) or 12 h (compound 1) prior to analysis by mass spectrometry on a Bruker 7 T Apex IV Fourier transform ion cyclotron resonance (FTICR) mass spectrometer. Samples were introduced to the mass spectrometer by electrospray ionization.

### Immunoprecipitation and glycosidase treatment

Lysates from doxycycline-induced T-REx™/FPN-V5 cells (370 μg of protein) were immunoprecipitated with anti-V5 antibody (Abcam) and protein G Dynabeads (Thermo Fisher). Immunoprecipitates were treated with PNGase F (New England BioLabs) according to New England BioLabs protocol with the following modifications: (1) denaturation at 50°C for 30 min and (2) PNGase F treatment for 3 h at 37°C. Samples were electrophoresed on a 4–20% Tris-Glycine gel, transferred to nitrocellulose and probed with Abs 38C8 (20 μg/ml) and 38G6 (1 μg/ml).

### Calculations, graphs, and statistics

GraphPad Prism® 6.07 was used for graph generation and analysis. Four-parameter variable slope non-linear regression was used for dose response curve fitting. Statistical significance was determined using an unpaired *t*-test and two-tailed *P*-values.

### Biohazards

All biological and chemical materials and procedures were performed in strict compliance with Amgen Environmental Health and Safety rules.

### Animal care

Mice and rats were housed in groups at an Association for Assessment and Accreditation of Laboratory Animal Care (AAALAC), International accredited facility. Animals were cared for in accordance with the *Guide for the Care and Use of Laboratory Animals*, 8th Edition. All research protocols were reviewed and approved by the Amgen Institutional Animal Care and Use Committee. Mice and rats (Charles River Laboratories) female, 6–8 weeks of age, were housed in individual ventilated caging (IVC) system on an irradiated corncob bedding (Envigo Teklad 7097). Lighting in animal holding rooms was maintained on 12:12 h light:dark cycle, and the ambient temperature and humidity range was at 68–79 F and 30–70%, respectively. Animals had *ad libitum* access to irradiated pelleted feed^2^ (Envigo Teklad Global Rodent Diet- soy protein free extruded 2020X) and reverse-osmosis (RO) chlorinated (0.3–0.5 ppm) water via an automatic watering system. Cages were changed biweekly inside an engineered cage changing station. For XenoMouse™: Research and all technical procedures performed on animals under this study were approved by the Animal Care Committee (ACC) for Amgen British Columbia. Animals were housed in a Canadian Council on Animal Care (CCAC) accredited facility and were cared for according to standards established by the CCAC and comply with institutional policies and guidelines. Animal experiments were executed in strict compliance with institutional guidelines and regulations.

## Results

### Highly specific anti-ferroportin antibodies were identified

Several previous studies have relied on expression of tagged FPN (e.g., GFP, MYC, FLAG) in engineered cells as the only means of detecting FPN, precluding the possibility of detecting endogenously-expressed protein. Tags such as MYC and FLAG encoded on the N- or C-termini of FPN also prevented detection in live cells, since both termini are intracellular (Liu et al., 2005; Rice et al., 2009; Wallace et al., 2010; Taniguchi et al., 2015). Anti-FPN antibodies are available from commercial sources, and some of these antibodies have been used in published studies to detect human FPN (Pinnix et al., 2010; Bonilla et al., 2012). We evaluated a number of these antibodies but we were unable to demonstrate that they detected human FPN (Supplementary Figure 1A), although one of these antibodies did detect mouse FPN (Supplementary Figure 1B). We generated anti-human FPN antibodies by immunizing mice (XenoMouse™ for fully human antibodies and C57BL/6 mice for mouse antibodies) with membranes from human FPN-expressing CHO cells. Hybridoma clones expressing antibodies that bound the extracellular region of FPN were selected by screening hybridoma supernatants against live human FPN-expressing 293T (T-REx™/FPN-V5) cells. The anti-human FPN antibodies discussed herein are 31A5, 45D8, 38C8, and 38G6 (Table 1). Anti-mouse FPN antibodies were generated by immunizing rats with membranes from mouse FPN-expressing CHO cells. Clones expressing antibodies that bound the extracellular region of mouse FPN were selected by screening hybridoma supernatants against live insect cells infected with a baculovirus-encoding mouse FPN. The anti-mouse FPN antibodies discussed herein are 1C7 and 1E11 (Table 1).

**Table 1:** Attributes of selected anti-FPN antibodies and tested applications.

**Antibody**	**Species**	**Epitope**	**Recognizes unglycosylated FPN**	**Tested application(s)**
1C7	Rat anti-mouse	IPETVF	Not tested	IB, IF, IHC
1E11	Rat anti-mouse	Not mapped	Not tested	IB, IF
31A5	Mouse anti-human	PETSP	Yes	IB, IF, FC, IHC
45D8	Mouse anti-human	Does not bind a linear epitope	Not tested	IF, FC
38G6	Human anti-human	IYMSNGSNS	Yes	IB, IF, FC
38C8	Human anti-human	Does not bind a linear epitope	No	IB, IF, FC

*IHC, immunohistochemistry; IB, immunoblot; IF, immunofluorescence (live and fixed/permeabilized cells); FC, flow cytometry*.

**Figure 1:**
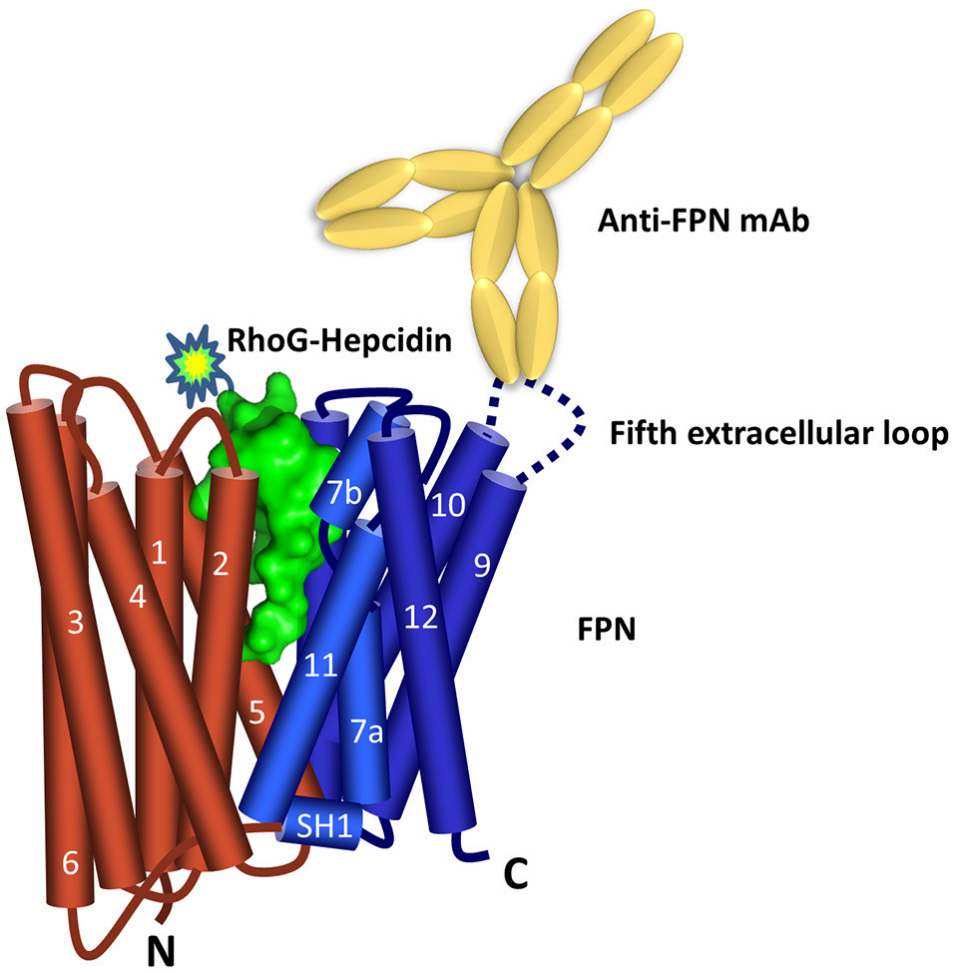
Ferroportin topology and binding sites. Predicted FPN (N lobe red and C lobe blue) topology (Taniguchi et al., 2015) includes 12 transmembrane domains with both N- and C-termini located inside the cell. Hepcidin surface model (green, derived from PDB 3H0T) is shown binding between the N and C lobes. The majority of the anti-FPN monoclonal antibodies recognize epitopes in the fifth extracellular loop.

Characterization of the binding epitope recognized by the FPN antibodies revealed that three of the six anti-human and anti-mouse antibodies (31A5, 38G6, 1C7) recognized sequences in the fifth extracellular (EC) loop (Figure 1). Sequence alignment shows that the amino acid sequence in the fifth extracellular loop between transmembrane helices 9 and 10 differs between human, mice and rats, whereas the regions on either side of this loop, containing residues important for binding hepcidin (including C326) are 100% conserved (Supplementary Figure 2). The sequence diversity in loop five may explain why the antibodies were primarily directed to this region.

**Figure 2:**
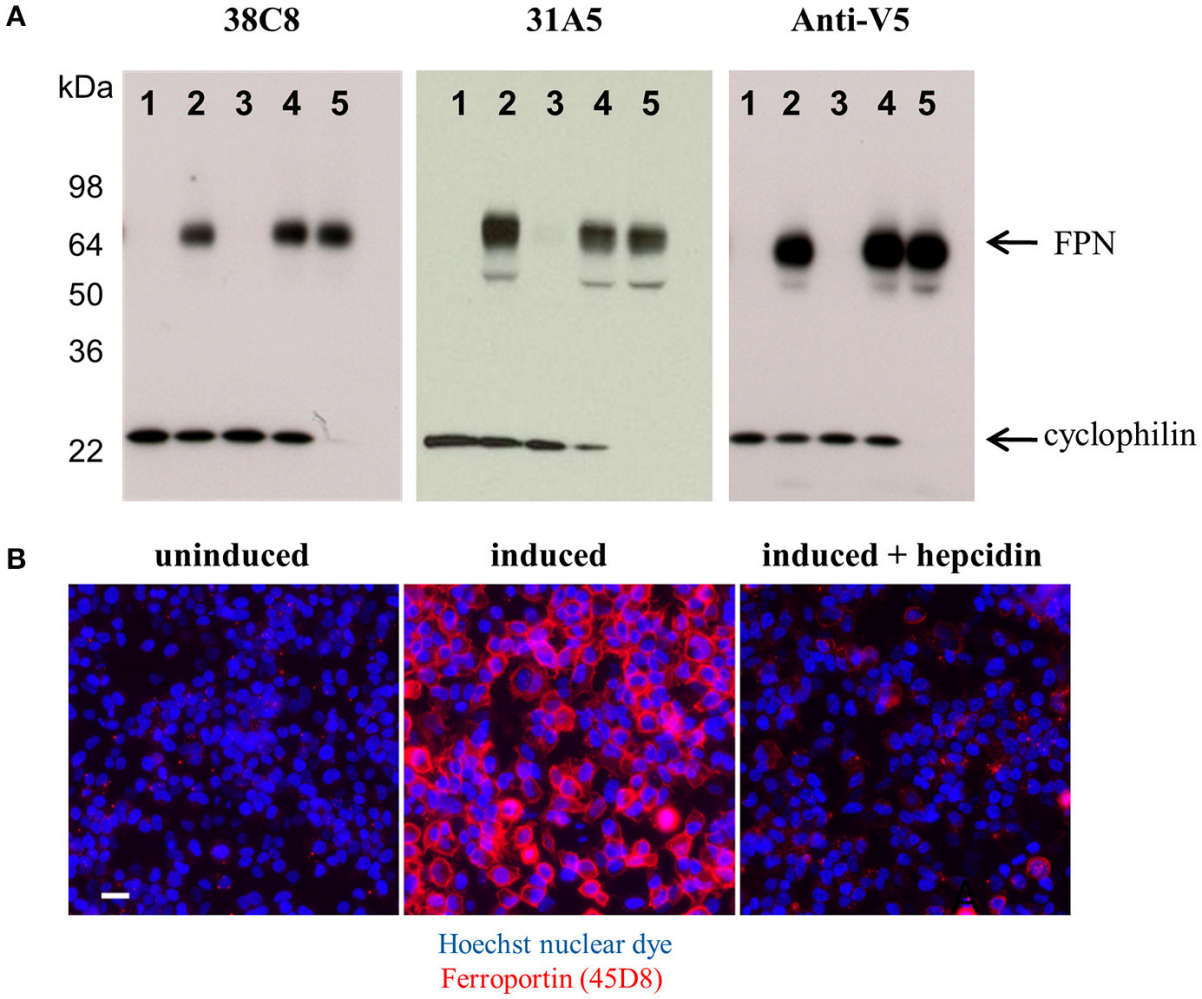
Anti-FPN monoclonal antibodies are specific for FPN. **(A)** T-REx™/FPN-V5 cells were treated with 80 nM siRNA reagents for 48 h. Lane 1, uninduced cells; lane 2, induced cells; lane 3, induced cells plus FPN siRNA; lane 4, induced cells plus non-silencing control siRNA; lane 5, induced cells plus cyclophilin siRNA. Blots were probed with either 10 ng/mL 38C8, 2 μg/ml 31A5 or 1 μg/ml anti-V5 antibodies and 0.5 μg/ml anti-CypB antibodies. **(B)** T-REx™/FPN-V5 cells were induced overnight and treated with 1 μM hepcidin for 2 h; fixed cells were stained with Hoechst nuclear dye (blue) and FPN was detected with Ab 45D8 (red). Scale bar = 20 μm.

Both the anti-mouse and -human FPN antibodies were capable of detecting FPN in multiple formats including immunoblotting, immunofluorescence, immunohistochemistry and flow cytometry. Table 1 provides a description of the antibodies tested and the applications for which they are suitable. Most of the antibodies were interchangeable with respect to applications, with few exceptions as noted in Table 1. Analogous data sets were generated for most of the antibodies discussed in this manuscript, and choice of using one antibody over another was often based on availability at the time of study. To characterize the antibodies, we frequently used engineered V5-tagged FPN-transfected T-REx™-293 cells (T-REx™/FPN-V5), where FPN expression was inducible by doxycycline (hereafter “doxycycline-induced” is referred to as “induced”). Antibody specificity was demonstrated by characterization of antibody binding to (1) uninduced and induced cells, (2) siRNA-treated induced cells and (3) hepcidin-treated cells. Western analysis results for two anti-human FPN antibodies (38C8 and 31A5), along with control anti-V5 antibody are shown in Figure 2A. FPN comprises 571 amino acids and contains 3 N-linked glycosylation sequences. On a non-reducing SDS-PAGE gel, FPN migrates at ~65 kDa. Antibodies 38C8 and 31A5 detect a band of ~65 kDa in lysates from induced cells and in lysates from induced cells treated with non-silencing siRNA control. Neither antibody is reactive with lysates from uninduced cells or cells treated with FPN siRNA. Since FPN has a V5 tag in these cells, an anti-V5 antibody (Abcam) was used as a positive control, which also detected a band of ~65 kDa. Based on these results we concluded that the 65 kDa band was FPN. Cyclophilin B (22 kDa) was used as a control for loading and to verify knockdown (Figure 2A). In addition to detecting FPN by Western analysis, it was also possible to detect surface-expressed FPN by immunofluorescence. As shown for anti-human FPN antibody 45D8, cell-surface FPN was detected on induced cells, but not on uninduced cells or on induced cells treated with hepcidin (Figure 2B).

### Anti-ferroportin antibodies detected endogenously-expressed ferroportin

**Figure 3:**
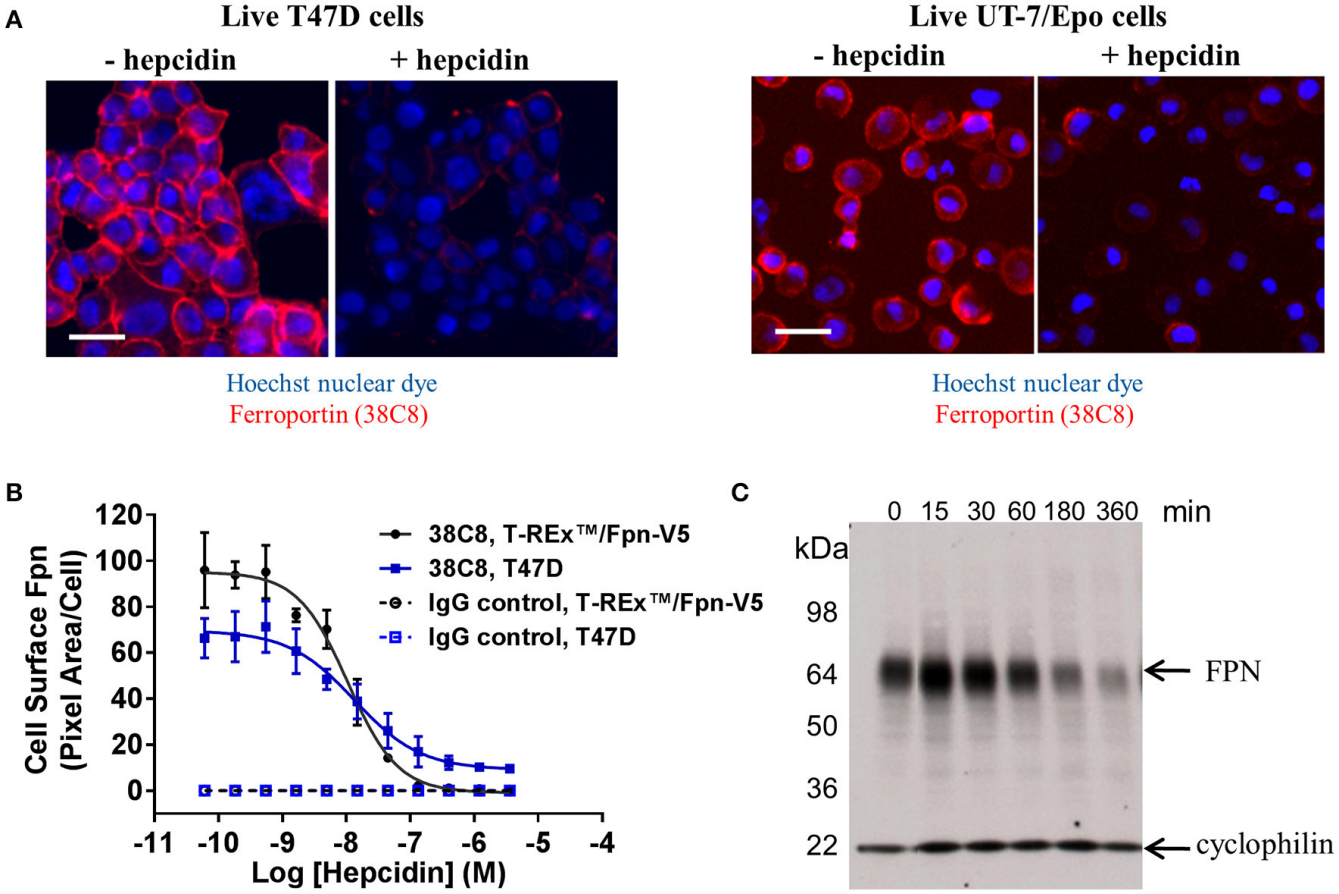
Anti-human ferroportin antibodies are suitable for detecting endogenously-expressed FPN. **(A)** T47D cells (left panel) and UT-7/Epo cells (right panel) were treated overnight with ferric citrate to increase FPN expression, then treated with 1 μM hepcidin for 6 h; FPN was detected on live cells with Ab 38C8 (red = FPN; blue = nuclei); scale bar = 20 μm. **(B)** T-REx™/FPN-V5 and T47D cells were treated with hepcidin for 6 h; surface FPN internalization was assessed on live cells by cellular imaging using Ab 38C8 (*N* = 4, mean ± sd). **(C)** UT-7/Epo cells were treated with hepcidin for the times shown; FPN was detected by Western analysis with Ab 38C8. Cyclophilin was used as a loading control.

We identified cell lines that endogenously express human FPN, including the ductal breast epithelial cell line T47D and the erythropoietin-dependent erythroleukemia cell line UT-7/Epo. Surface FPN was detectable using the anti-human FPN antibodies described above and this expression could be increased by treatment with ferric citrate (not shown) and decreased in response to treatment with hepcidin (Figure 3A). Using these antibodies, it was possible to develop a quantitative cellular imaging assay that measured hepcidin-induced FPN internalization. Data from this assay indicated that T47D cells responded to hepcidin comparably to the engineered T-REx™/FPN-V5 cell line, with EC_50_ values for FPN internalization of 13 nM and 11 nM, respectively, after 6 h of treatment (Figure 3B). Hepcidin-mediated FPN degradation in UT-7/Epo cells was corroborated by Western analysis where the FPN signal had nearly disappeared after 6 h (Figure 3C).

**Figure 4:**
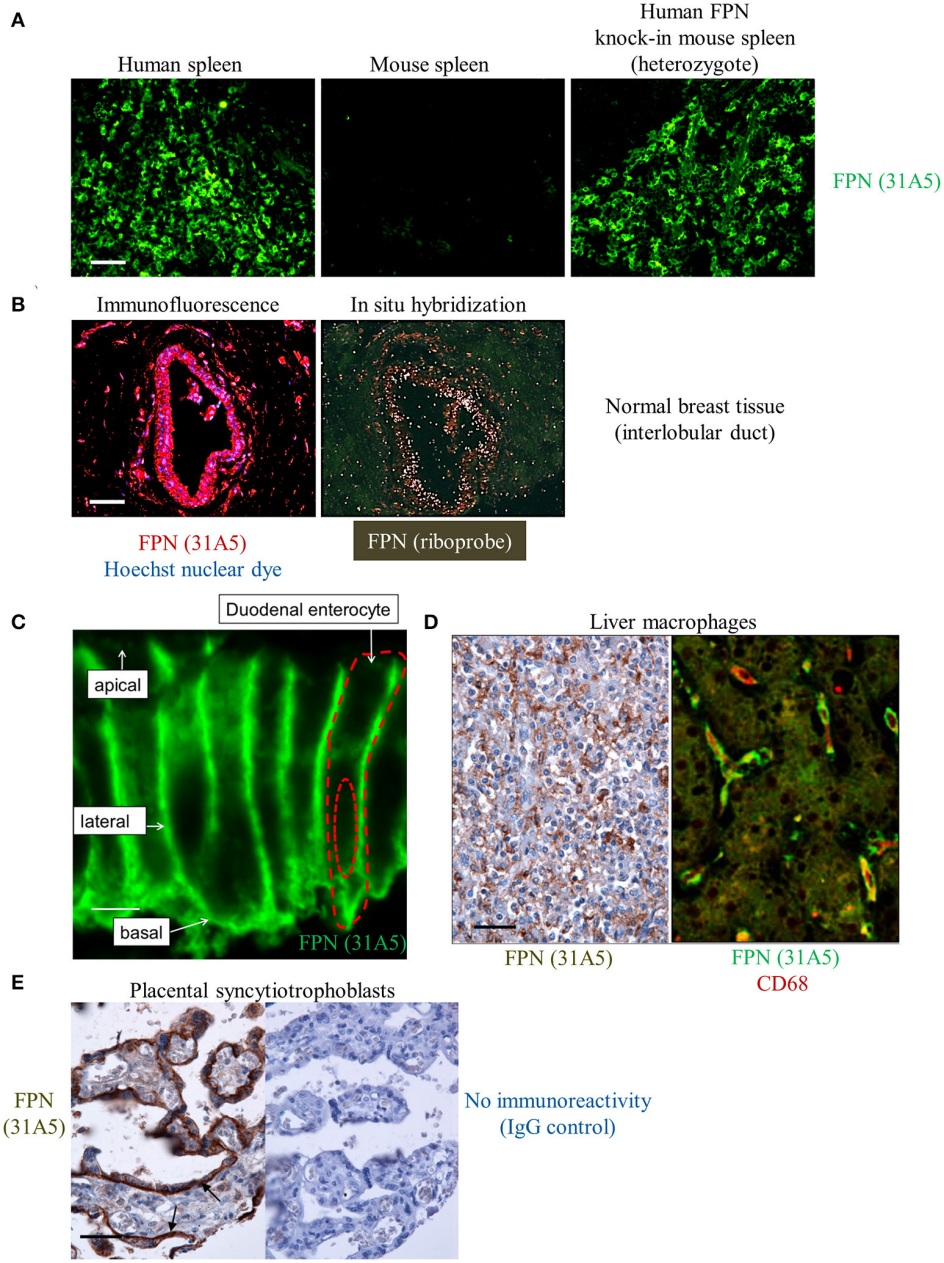
Anti-human ferroportin antibody detects FPN in tissue with specificity and sensitivity. **(A)** Human FPN was detected using direct immunofluorescence in formalin-fixed human and mouse spleen tissues using Ab 31A5-FITC; scale bar = 50 μm. **(B)** Human FPN was detected in formalin-fixed normal human breast tissue using Ab 31A5 (left panel, red = FPN; blue = DAPI). FPN mRNA was detected in serial tissue sections by isotopic (^33^P) *in situ* hybridization (right panel) using an antisense riboprobe directed against a portion of human FPN; scale bar = 50 μm. **(C)** Duodenum at high magnification showing basolateral FPN expression (green); scale bar = 5 μm. **(D)** FPN staining in liver macrophages in hepatic sinusoids (brown, left panel) and FPN (green) colocalizing with macrophage marker CD68 (red) in Kupffer cells (right panel); scale bar = 50 μm. **(E)** Placental tissue stained with Ab 31A5 (brown, left panel) or isotype control (right panel); arrows point to basal immunoreactivity in syncytiotrophoblasts; scale bar = 50 μm.

Endogenous human FPN expression was also detectable by immunohistochemistry (IHC), demonstrated using antibody 31A5. This antibody recognizes a sequence in human FPN that is not conserved in mouse FPN. To demonstrate specificity for human FPN, antibody 31A5 was tested on human spleen, mouse spleen and mouse spleen in which human FPN had been knocked in. As shown in Figure 4A, antibody 31A5 produced a robust signal in human spleen and in the mouse spleen expressing human FPN; however, it did not produce a signal in mouse spleen. Specificity was further confirmed in human breast tissue where FPN detection by IHC was corroborated by *in situ* hybridization (ISH, Figure 4B). FPN expression has been reported in the duodenum, reticuloendothelial macrophages and placenta of mice (Abboud and Haile, 2000; Donovan et al., 2005). We investigated FPN expression in a panel of human tissues. In the duodenum, FPN expression was confirmed on the basolateral surface of enterocytes (Figure 4C, Donovan et al., 2000). FPN expression was detected in Kupffer cells of the liver (Figure 4D) and in interstitial mononuclear cells from esophagus, stomach, intestine, kidney, prostate, cervix, and skin (not shown). FPN was also detected in syncytiotrophoblasts of the placenta (Figure 4E).

**Figure 5:**
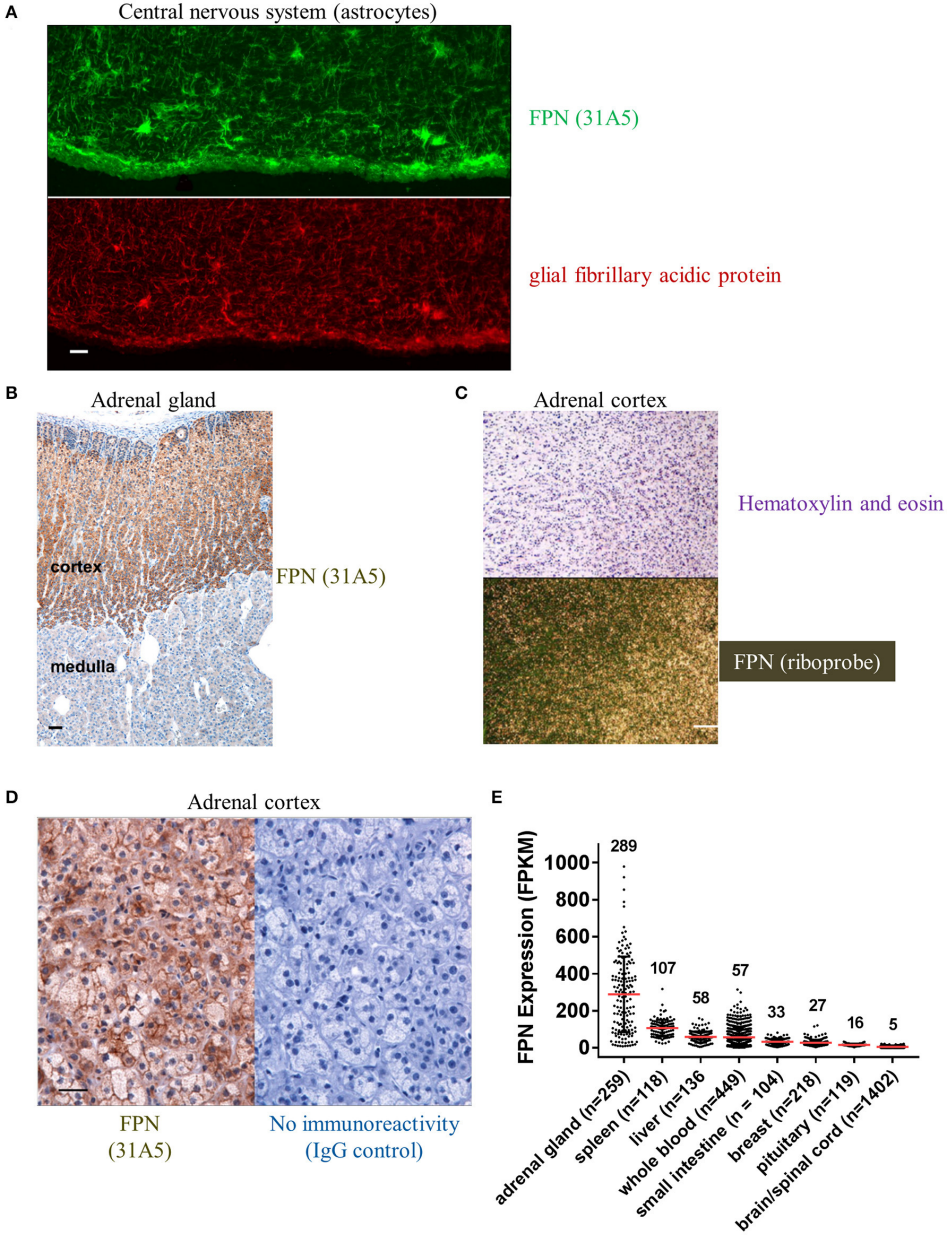
Ferroportin is detected in the CNS and adrenal cortex. **(A)** FPN detected in the CNS with astrocytes showing robust staining (green, top panel) that colocalizes with the astrocyte marker glial fibrillary acidic protein (GFAP, red, bottom panel); scale bar = 50 μm. **(B)** Robust FPN staining observed with Ab 31A5 in adrenal cortex (brown), but not medulla; scale bar = 50 μm. **(C)** FPN expression in adrenal cortex was confirmed by *in situ* hybridization (top panel, H&E stain; bottom panel, ISH with FPN probe; scale bar = 200 μm). **(D)** Adrenal cortex showing FPN localization to cell surface (Ab 31A5, left panel; isotype control, right panel); scale bar = 50 μm. **(E)** FPN mRNA expression levels in multiple tissues were determined by RNA sequencing and expressed as fragments per kilobase of exon per million fragments mapped (FPKM). Mean expression levels (red lines, values shown above plot) and number of tissues sequenced (n) are shown. Data used for analysis were obtained from the Genotype Tissue Expression (GTEx) Project on 02/28/2017.

We also detected FPN in tissues for which expression has not been previously described. Antibody 31A5 detected expression in neuronal astrocytes (Figure 5A), and robust expression of FPN was detected in the cortical, but not medullary cells of the adrenal gland (Figure 5B). FPN expression in adrenal cortex was confirmed by ISH (Figure 5C), and was localized to the membrane (Figure 5D), suggesting its function as an iron exporter is preserved in this tissue. Expression in adrenal gland is corroborated by RNA data from the Genotype Tissue Expression (GTEx) Project database for RNA sequencing data where adrenal gland is the tissue with the highest mean FPN mRNA content of all tissues characterized (Figure 5E). It is unclear why FPN is so highly expressed in the adrenal cortex.

### Fluorescently-labeled hepcidin analog induced ferroportin internalization with potency similar to wild type

**Figure 6:**
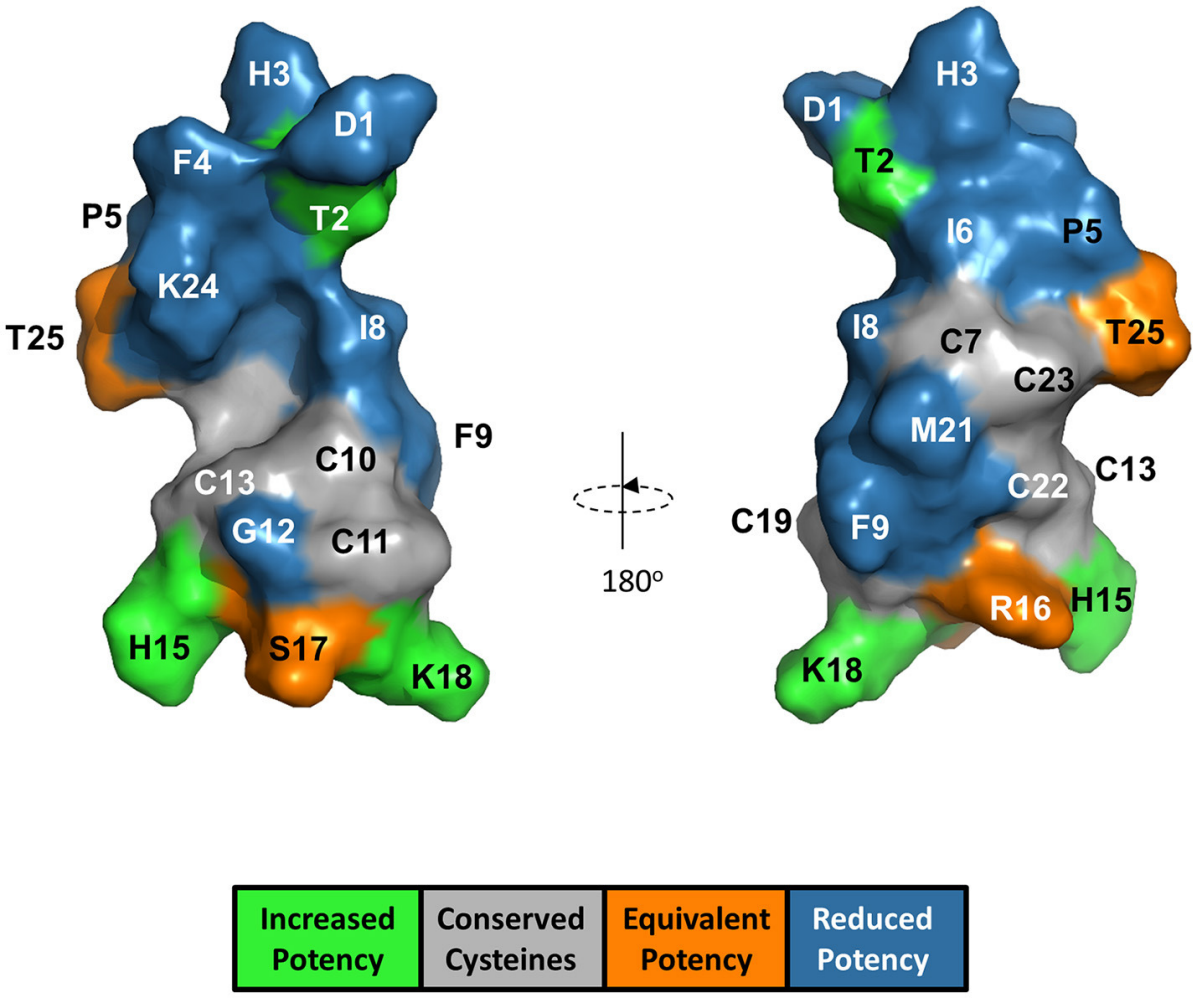
Alanine substitution of non-cysteine hepcidin residues identified positions important for hepcidin activity and positions suitable for label derivatization. Hepcidin structure (PDB 2KEF) is overlaid with alanine mutant potency data. G20 is not visible from the surface.

**Figure 7:**
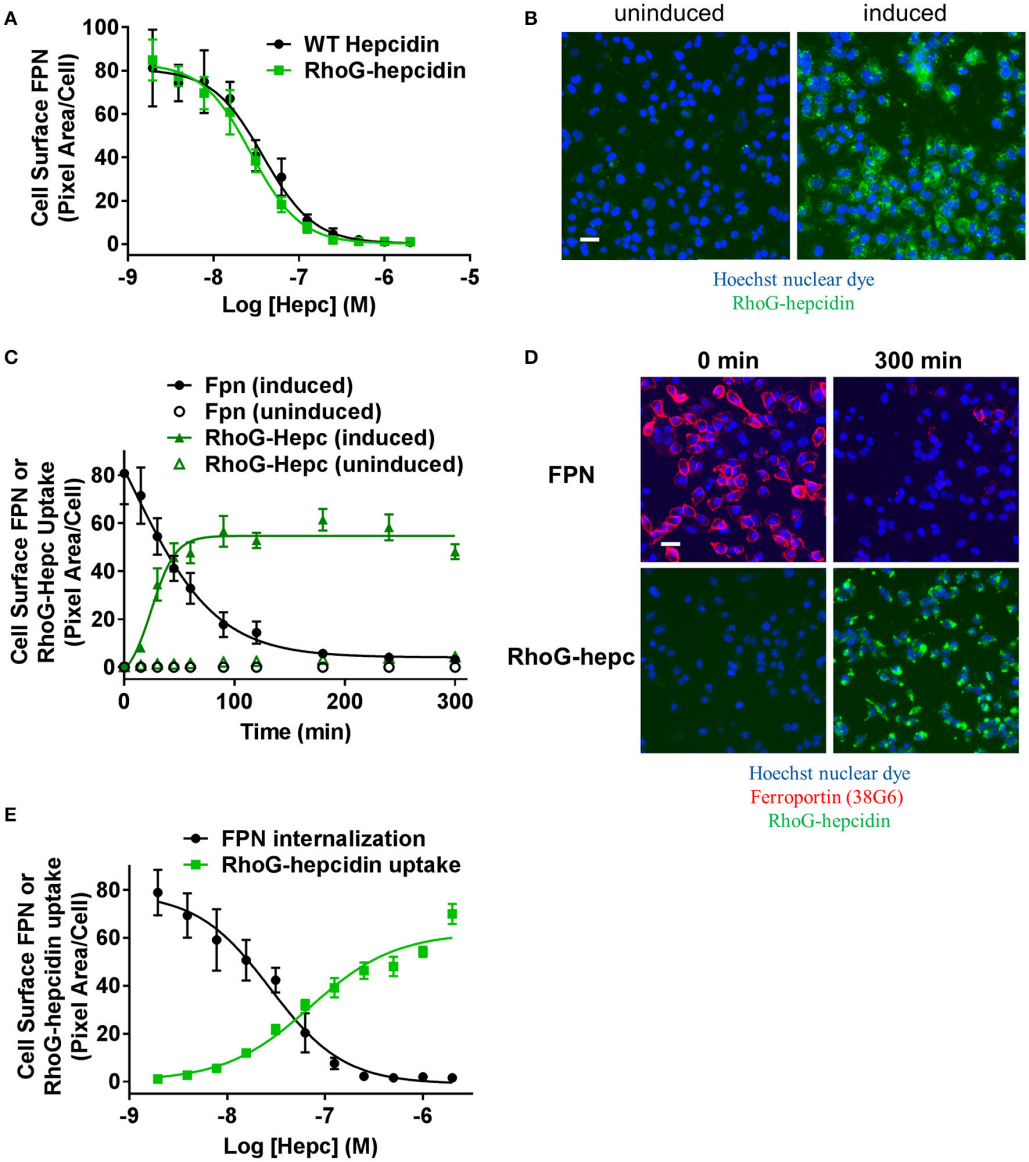
Fluorescently-labeled hepcidin analog induces ferroportin internalization with potency similar to wild type. **(A)** T-REx™/FPN-V5 cells were induced and treated with a dose range of RhoG-hepc or unlabeled hepcidin for 3 h. Live cells were stained on ice with Ab 38C8 and FPN internalization was measured with cellular imaging (*N* = 4, mean ± sd). **(B)** Representative images detecting RhoG-hepcidin uptake for uninduced and induced T-REx™/FPN-V5 cells treated for 1 h with 300 nM RhoG-hepc; scale bar = 20 μm. **(C)** Uninduced or induced HEK-FPN-V5 cells were treated with RhoG-hepc for 0–300 min with 250 nM RhoG-hepc; FPN internalization (live cells, Ab 38G6) and RhoG-hepcidin uptake were measured with cellular imaging (*N* = 4, mean ± sd). **(D)** Representative images of induced HEK-FPN-V5 cells showing FPN internalization and RhoG-hepc uptake at 0 and 300 min after RhoG-hepc treatment; scale bar = 20 μm. **(E)** Dose response curves for loss of cell surface FPN detected with Ab 38C8 and RhoG-hepcidin uptake after 3 h treatment with RhoG-hepcidin (*N* = 4, mean ± sd).

The anti-FPN antibodies provided a means of monitoring changes in FPN expression. To develop a trackable form of hepcidin, we initially labeled the N-terminus with Rhodamine green (RhoG) using linkers of different lengths; however, all N-labeled forms of hepcidin were inactive (not shown), corroborating previously-published work identifying the N-terminus as necessary for binding to FPN (Nemeth et al., 2006). We then initiated a campaign to synthesize hepcidin analogs that were amenable to labeling at positions other than the N-terminus. Hepcidin is a tightly-folded peptide containing four disulfide bonds that are conserved across species from fish to mammals (Shike et al., 2004; Jordan et al., 2009). We used alanine scanning to identify a derivatization point for the label by individually replacing all non-cysteine amino acids with alanine and testing them for bioactivity in an assay that monitors surface FPN expression (FPN internalization assay) and an iron response reporter assay. Briefly, the iron response assay detects intracellular iron accumulation through the expression of a reporter controlled by an iron response element (IRE) (Li et al., 2004). T-REx™/FPN-V5 cells were engineered with a β-lactamase (BLA) reporter gene downstream of the ferritin IRE, and BLA activity was detected by ratiometric fluorescence resonance energy transfer (FRET) upon addition of substrate. We found most substitutions resulted in reduced activity when compared to wild type (WT), with hydrophobic and aromatic residues in the N-terminal portion of the molecule having the most pronounced effect (I8, F4, I6, P5, F9, and H3). Substitutions at H15, K18, and T2 resulted in increased activity, while substitutions at residues R16, S17, and T25 were roughly equipotent to WT hepcidin (Table 2). Given the current model of the hepcidin/FPN interaction, where residues 16 and 17 are predicted to be pointing away from the complex (Taniguchi et al., 2015), it is not surprising that these residues could be labeled and still maintain the ability to bind FPN. We mapped the outcome of the alanine mutagenesis activity data on the crystal structure (Figure 6, Jordan et al., 2009) and selected S17 for derivatization as this residue is distal from the N-terminus which is reported to be necessary for FPN binding, and replacement of serine with RhoG labeled lysine (neutral charge) would not change the overall charge of the molecule. We tested linkers of different molecular weights and selected one of the lowest molecular weight linkers, comprising a single amino hexanoic acid (Ahx) molecule as analogs with longer linkers were inactive (data not shown). The final analog that we refer to as RhoG-hepcidin is [Lys17(*N*^ε^*-*Rhodamine green amino hexanoyl)]hepcidin. We found that the labeled analog was similar in potency to WT unlabeled hepcidin when measuring FPN internalization by cellular imaging (Figure 7A), EC_50_~30 nM in this assay. As expected, RhoG-hepcidin uptake was apparent in induced, but not uninduced cells (Figure 7B) indicating that interaction with FPN was required for hepcidin internalization. Using RhoG-hepcidin, it was possible to concurrently monitor hepcidin uptake and FPN internalization. Quantification of these signals demonstrated they were well correlated with respect to kinetics (Figures 7C,D) and potency (Figure 7E).

**Table 2:** Hepcidin analogs with alanine mutations tested in two different assays.

**Hepcidin analog**	**Iron response assay EC_50_ (nM)**	**Iron response assay mutant/WT EC_50_**	**FPN internalization assay EC_50_ (nM)**	**FPN internalization assay mutant/WT EC_50_**	**Mean mutant/WT EC_50_**
WT	63	–	51	–	–
H15A	12	0.2	10	0.2	0.2
K18A	13	0.2	12	0.2	0.2
T2A	29	0.5	29	0.6	0.5
T25A	53	0.8	39	0.8	0.8
R16A	57	0.9	56	1.1	1.0
S17A	66	1.0	50	1.0	1.0
M21A	112	1.8	162	3.2	2.5
G12A	107	1.7	212	4.2	2.9
D1A	144	2.3	185	3.6	3.0
K24A	266	4.2	267	5.2	4.7
G20A	288	4.6	309	6.1	5.3
H3A	298	4.7	308	6.0	5.4
F9A	392	6.2	250	4.9	5.6
P5A	180	2.9	474	9.3	6.1
I6A	634	10.1	247	4.8	7.5
F4A	>3,600	–	>3,600	–	–
I8A	>3,600	–	>3,600	–	–

*Mean values for both assays (N = 3 replicates)*.

### Antibodies were partially protective against hepcidin-mediated ferroportin internalization

**Figure 8:**
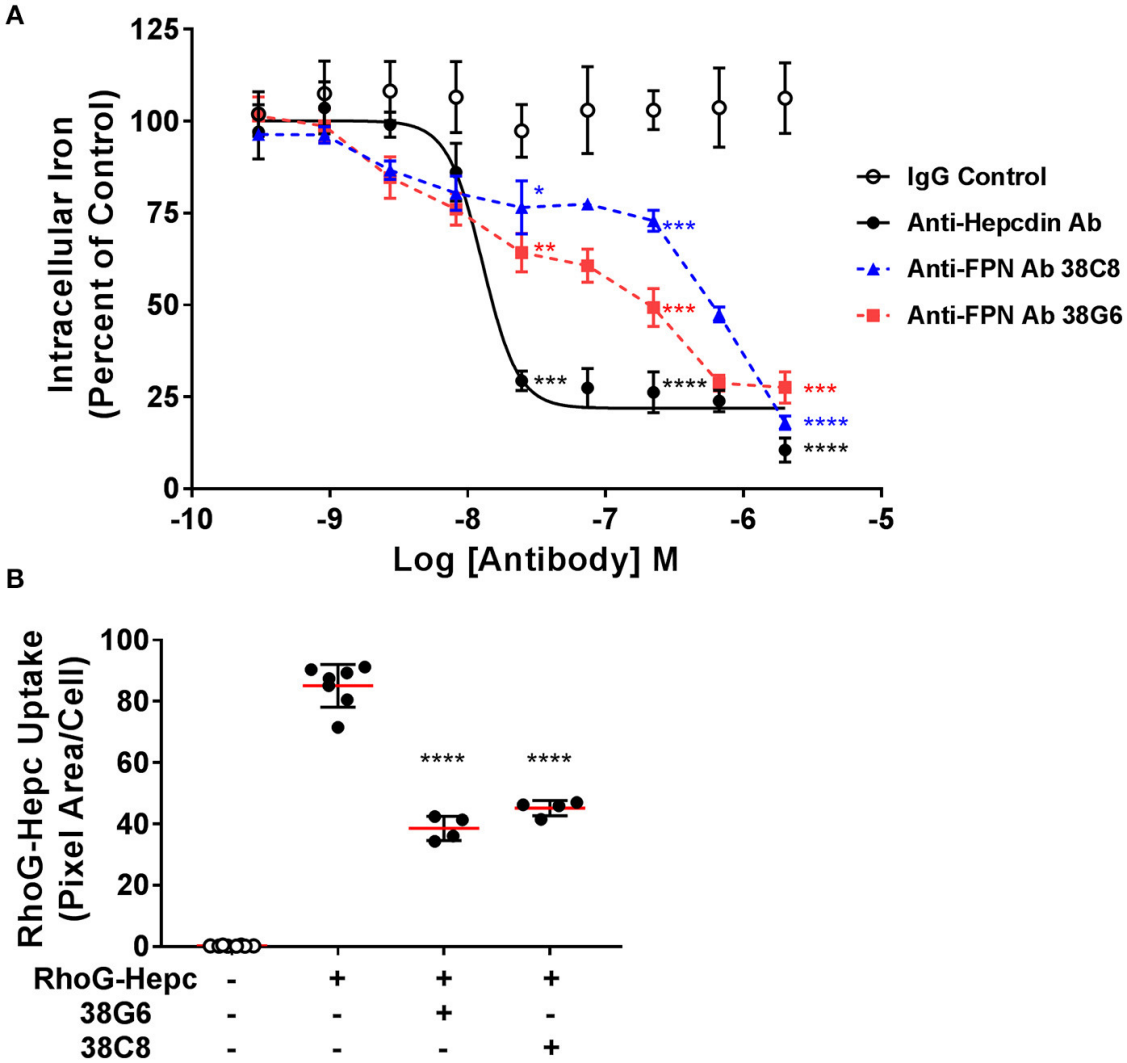
Anti-ferroportin antibodies were partially protective against hepcidin-mediated ferroportin internalization. **(A)** T-REx™FPN-V5/BLA cells were induced and treated with a dose range of antibody for 1 h prior to addition of 36 nM hepcidin for 18 h. Iron response percent of control was calculated from FRET ratio (*N* = 3, mean ± sd). **(B)** T-REx™/FPN-V5 cells were treated with 1.5 μM antibody for 1 h, followed by 250 nM RhoG-hepcidin for 1.5 h. RhoG-hepcidin uptake was measured by quantitative cellular imaging (*N* = 4–8, mean ± sd). Significance values: ^*^P < 0.05, ^**^P < 0.01, ^***^P < 0.001, ^****^P < 0.0001, compared to IgG untreated control (unpaired *t*-test and two-tailed *P*-values).

As described above, epitope mapping of the anti-FPN monoclonal antibodies revealed that antibodies either recognized linear sequences in the fifth EC loop of FPN, or they appeared to bind a conformational epitope (Table 1). The structure of FPN, based on the structure of the bacterial FPN homolog, BbFPN (Taniguchi et al., 2015), is proposed to comprise two transmembrane lobes and hepcidin is proposed to bind within the central cavity formed by these lobes (Figure 1). Because the fifth loop is not predicted to be involved in hepcidin binding, we did not anticipate that antibodies binding to the fifth EC loop would directly antagonize hepcidin binding; however, it was theoretically possible that the antibodies might stabilize FPN through a different mechanism. To determine if the monoclonal antibodies were capable of preserving FPN on the cell surface in the presence of hepcidin, we evaluated their activity using two different methods: iron response reporter (BLA) and RhoG-hepcidin uptake assays. Iron reporter cells were incubated with titrated anti-FPN antibodies 38C8 or 38G6, or control antibodies (IgG or anti-hepcidin; Sasu et al., 2010) for 1 h prior to addition of hepcidin at ~EC_70_ (36 nM) for 18 h. Although the anti-FPN antibodies showed activity that was significantly different than control, they were not as effective as the anti-hepcidin positive control antibody at maintaining surface FPN expression. In this assay, the maximum activities of tested antibodies were ~700 nM for antibody 38G6, ~2 μM for antibody 38C8 and ~25 nM for the anti-hepcidin antibody (Figure 8A). The RhoG-hepcidin uptake assay is a more proximal assay than the iron response assay, but the optimal RhoG-hepcidin concentration required for detection (250 nM) is higher than the EC_70_ value (36 nM) used in the iron response reporter assay. Cells were pre-incubated with antibodies for 1 h before addition of 250 nM RhoG-hepcidin. RhoG-hepcidin uptake was then measured 1.5 h later. Once again, partial protection was observed as Abs 38C8 and 38G6 reduced internalization by roughly 50% (Figure 8B). The protective effect was observed to be reduced with higher concentrations of hepcidin, although the concentrations used in both assays may exceed those observed in some diseased states (Ganz et al., 2008; Zaritsky et al., 2009; Macdougall et al., 2010).

### RhoG-hepcidin and monoclonal antibodies enabled small molecule screening assays

**Figure 9:**
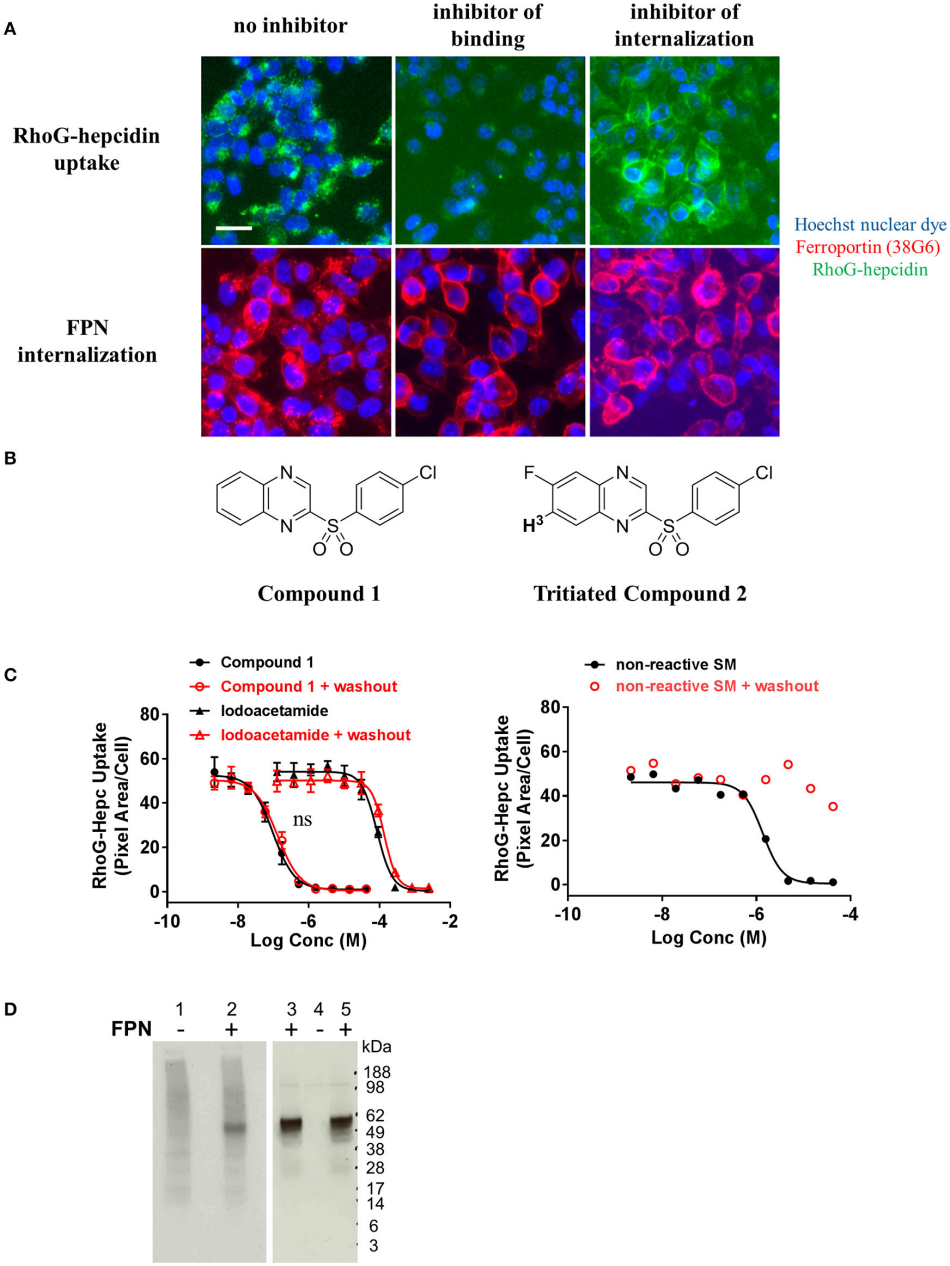
Small molecules that inhibit ferroportin internalization were identified. **(A)** T-REx™/FPN-V5 cells were induced overnight and treated with small molecules for 1 h prior to addition of RhoG-hepc for 2 h. Cells were fixed/permeabilized and stained with Ab 38G6; blue = nuclei, green = RhoG-hepcidin, red = FPN. Scale bar = 20 μm. Compounds were identified that inhibited hepcidin binding to FPN (middle panels) and inhibited FPN internalization (right panels). **(B)** Structures of Compound 1 and tritiated Compound 2. **(C)** T-REx™/FPN-V5 cells were incubated with compound **1** or iodoacetamide (left panel) or a reversibly-binding compound (right panel) for 1 h, followed by ± washing 2X with PBS and incubation with RhoG-hepcidin for 1 h. RhoG-Hepc uptake was measured by cellular imaging (*N* = 3, mean ± sd, left panel; *N* = 1 representative assay, right panel). ns, *P* > 0.05, comparing ± washout (unpaired *t*-test and two-tailed *P*-values). **(D)** Lysates from T-REx™/FPN-V5 cells treated with tritiated compound **2** were analyzed by non-reducing SDS-PAGE for ^3^H detection (left panel) or FPN detection by Western analysis with Ab 31A5 (right panel). Uninduced cells (lanes 1, 4) and induced cells (lanes 2, 3, 5) were incubated with 330 nM ^3^H-Compound 2 (lanes 1, 2, 4, 5) or dimethyl sulfoxide, compound diluent (DMSO, lane 3) for 30 min.

In an effort to identify small molecule inhibitors of hepcidin-induced FPN internalization, we ran a small molecule screen using the cell-based iron response reporter assay described above. A library of ~300,000 small molecules was screened in a single-point assay and hits were titrated to determine their potency. Molecules that prevented intracellular iron accumulation were further tested in the RhoG-hepcidin uptake and FPN internalization assays. The uptake assay indicated that the small molecules had different modes of action; some compounds inhibited FPN internalization by blocking hepcidin binding and others inhibited FPN internalization without directly blocking hepcidin (Figure 9A). Compounds from the latter phenotypic group were found to be non-specific, as they were (1) active in a general endocytosis counterscreen and/or (2) analogs of the compounds were found to be inactive. One compound that inhibited RhoG-hepcidin uptake was a sulfonyl quinoxaline (Compound **1**, IC_50_ = 141 nM, Figure 9B). Activated heteroaromatic molecules like 2-sulfonyl quinoxalines can interact with nucleophiles (Sundaram et al., 2007); hence we evaluated the reactivity of compound **1** in the presence of biologically-relevant nucleophiles. Incubation of compound **1** and related 2-sulfonyl quinoxalines in our collection with glutathione showed formation of a glutathione adduct (data not shown). FPN contains a cysteine (C326) in the fourth loop that is reported to contain a free sulfur, i.e., not involved in a disulfide bond (Liu et al., 2005). Previous reports by Nemeth et al. (Fernandes et al., 2009) indicate that treatment of FPN with non-membrane permeable thiol-modifying reagents such as iodoacetamide prevents hepcidin binding, presumably through reaction with the free sulfur in C326. However, hepcidin also contains eight cysteines and while all of these cysteines are reported to be involved in disulfide bonds (Jordan et al., 2009), it is theoretically possible that the compounds could react with hepcidin if the disulfide bonds were labile. To determine whether the hepcidin-blocking effect of compound **1** was reversible, we tested compound **1** and iodoacetamide in a modified version of the RhoG-hepcidin uptake assay. In this instance we treated cells with compound **1** or iodoacetamide for 1 h and then washed the cells to remove any unbound compound; RhoG-hepcidin was then added and uptake assessed. In this format, the positive control iodoacetamide blocked RhoG-hepcidin binding with an IC_50_ of 86 μM, while compound **1** was active at a significantly lower concentration with IC_50_ value of 0.1 μM. Similar potencies were observed in experiments in which compound was continuously present (Figure 9C, left panel). These data indicate that the compounds bound irreversibly to target, in contrast to a control non-reactive, reversibly-binding compound, which was inactive after washout (Figure 9C, right panel). We confirmed the compounds bound irreversibly to FPN using a tritium-labeled analog of compound **1** (compound **2**, Figure 9B). Compound **2** was incubated with uninduced and induced T-REx™Fpn-V5 cells and was only detectable in lysates from induced cells. Association of tritiated compound **2** with FPN was established by demonstrating that the compound **2**-labeled band migrated at the same molecular weight as FPN, detected by Western analysis (Figure 9D).

**Figure 10:**
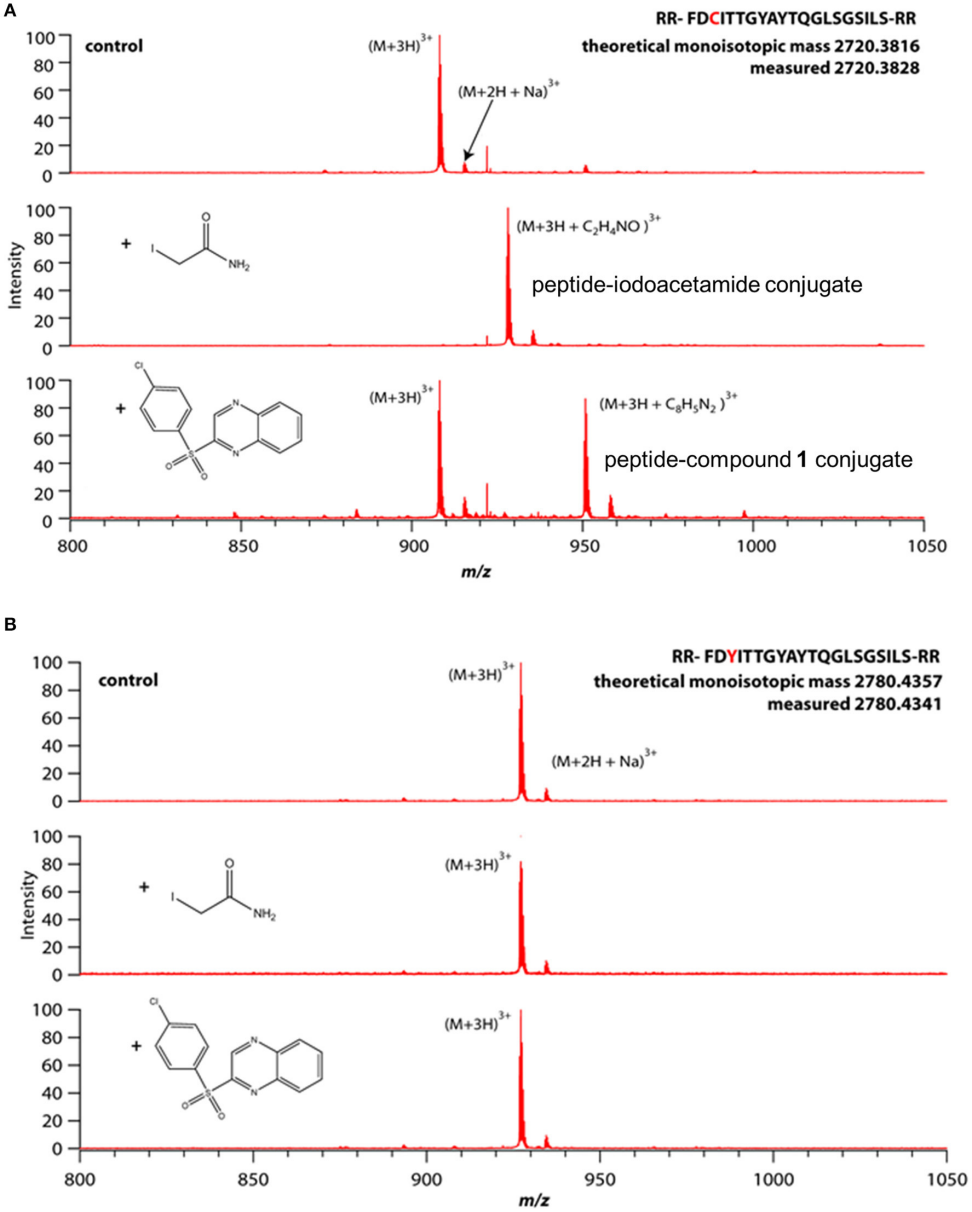
Compound **1** reactivity with linear peptide requires cysteine thiol. Peptides were incubated with small molecules (100 μM each) in 20 mM acetate buffer, pH 7 for 1 h (iodoacetamide) or 12 h (Compound **1**) prior to analysis by mass spectrometry. **(A)** Peptide comprising WT hepcidin binding domain (HBD) containing an unpaired cysteine. **(B)** Mutant HBD with cysteine replaced by tyrosine.

The thiol reactivity of compound **1** was demonstrated using two linear peptides; one composed of the FPN sequence including C326 which is required for hepcidin binding (Figure 10A); the other was composed of the same sequence with the exception that C326 was replaced with the non-thiol amino acid tyrosine (Figure 10B). Adduct formation was assessed with mass spectrometry, using iodoacetamide as a control. Reactivity of compound **1** with the peptide was demonstrated by observation of adducts corresponding to the incorporation of the quinoxaline core via substitution of the sulfonyl moiety and was only observed when cysteine was present (Figure 10). We inferred that compound **1** potentially reacted with C326 in cell-expressed FPN.

To evaluate the selectivity of compound **1** for folded FPN vs. thiol reactivity, we compared the rate of reaction of compound **1** with cell-expressed FPN vs. the rate of reaction with the C326-containing linear peptide. A faster rate of reaction with cellular FPN vs. the linear peptide would suggest that compound **1** has some affinity for folded FPN and binding facilitates reaction with the thiol moiety of C326. Reactivity with cell-based, folded FPN was assessed by the hepcidin uptake assay (Figure 9C); reactivity with the linear peptide was assessed by mass spectrometry (Figure 10A). In cells, 100 μM compound **1** inhibited 100% of hepcidin uptake after just 1 h. In comparison, 100 μM compound **1** reacted with only 50% of the peptide in 12 h. This suggests that compound **1** has some affinity for folded FPN protein. Iodoacetamide, which has no affinity for folded FPN and was selected as a control because of its thiol reactivity, reacted completely with the cysteine-containing peptide within 1 h (100 μM iodoacetamide) whereas this same concentration only inhibited ~50% of hepcidin uptake within the same time frame.

### Reagents extended understanding of ferroportin-hepcidin interaction

**Figure 11:**
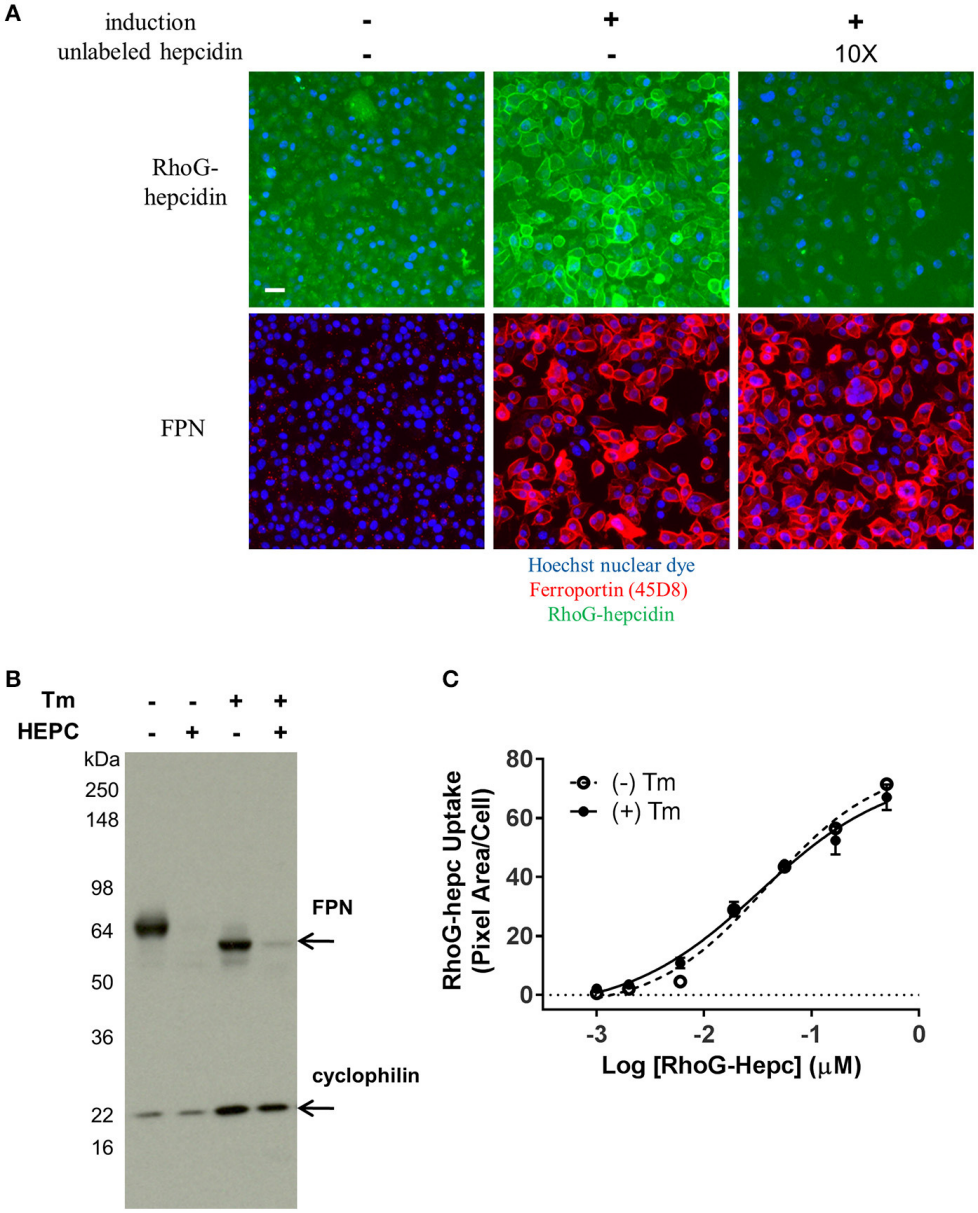
FPN binds RhoG-hepcidin reversibly and does not require N-linked glycosylation for hepcidin binding or internalization. **(A)** T-REx™/FPN-V5 cells were allowed to equilibrate on ice for 15 min prior to addition of RhoG-hepcidin for 2 h on ice; 10X unlabeled hepcidin was added for 2 additional hours on ice. FPN was detected with Ab 45D8; scale bar = 20 μm. **(B)** Non-glycosylated FPN binds hepcidin and internalizes. T-REx™/FPN-V5 cells were treated ± 0.3 μg/ml tunicamycin (Tm) for 18 h, followed by treatment with 500 nM hepcidin (Hepc) for 5 h. Blot was probed with anti-V5 Ab for FPN detection and anti-cyclophilin as loading control. **(C)** Induced HEK-FPN-V5 cells were treated ± 0.25 μg/ml tunicamycin (Tm) for 18 h, followed by RhoG-hepcidin for 3 h; uptake was quantitated by cellular imaging (*N* = 4, mean ± sd).

It was previously reported that mammalian hepcidin did not bind to FPN at low temperatures (De Domenico et al., 2008). To verify this work, RhoG-hepcidin was incubated with either uninduced or induced cells at 0°C. Contrary to the previous report, RhoG-hepcidin bound to FPN at this low temperature. To assess the reversibility of hepcidin binding to FPN, we incubated FPN-expressing cells with RhoG-hepcidin at 0°C to prevent internalization; we then added excess unlabeled hepcidin to determine if the labeled hepcidin could be displaced. We found that excess unlabeled hepcidin effectively competed pre-bound RhoG-hepcidin from the surface of FPN-expressing cells under conditions where FPN/hepcidin could not be internalized, i.e., at 0°C. We confirmed that FPN was not internalized using antibody 45D8 (Figure 11A). These results demonstrated that RhoG-hepcidin bound FPN at 0°C and that the binding was reversible.

FPN has three potential N-linked glycosylation sites (N174, N434 and N567). One of these sites (N434) is in the fifth EC loop, the same loop to which our monoclonal antibodies bind. Tissue-specific differences in hepcidin activity in mice have been reported, and it was suggested that FPN glycosylation or other post-translational modifications may be responsible for these differences (Canonne-Hergaux et al., 2006; Drakesmith et al., 2015). To determine if glycosylation of human FPN is required for hepcidin-mediated internalization and degradation, we treated induced T-REx™/Fpn-V5 cells with tunicamycin, which blocks addition of N-linked glycosylation (Lehle and Tanner, 1976); cells were subsequently treated with hepcidin, which resulted in degradation of both glycosylated and non-glycosylated FPN, as shown by Western blot (Figure 11B). Similarly, we saw no difference in RhoG-hepcidin uptake with and without tunicamycin treatment (Figure 11C). We also discovered that one of our human monoclonal antibodies, antibody 38C8, did not recognize enzymatically deglycosylated FPN (Peptide-N-Glycosidase F (PNGase F)-treated). Western analysis revealed that antibody 38G6 recognized both glycosylated and deglycosylated FPN, but PNGase F-treated FPN was not detectable with antibody 38C8, even at the very high concentration of 20 μg/ml (Supplementary Figure 3A). When cells were treated with tunicamycin followed by hepcidin, we confirmed by immunoblot that (1) antibody 38G6, but not antibody 38C8, detected non-glycosylated FPN and (2) hepcidin-mediated FPN internalization did not require N-linked glycosylation of FPN (Supplementary Figure 3B). Detection of non-glycosylated FPN was also observed with antibody 38G6, but not antibody 38C8, following tunicamycin treatment in immunofluorescence experiments (Supplementary Figure 3C).

## Discussion

We sought to identify a FPN antagonist capable of maintaining FPN iron export in the presence of elevated hepcidin concentrations, as occurs in chronic inflammatory conditions. We started by generating reagents needed for proximal, high fidelity cell-based assays. We developed mouse anti-human FPN monoclonal antibodies and screened them against human FPN-transfected cells where expression was inducible. This provided a convenient means for assessing antibody specificity. We selected antibodies that bound to live induced, but not uninduced T-REx™/Fpn-V5 cells. We used these antibodies (e.g., 45D8, 31A5) to follow the loss of cell-surface FPN upon treatment with hepcidin in quantitative image-based assays, and to elucidate FPN expression in a panel of human tissues. Expression was observed in expected (enterocytes, macrophages, placenta) and unexpected (astrocytes, adrenal cortex) tissues. Concurrently, we generated fully human anti-human FPN antibodies (e.g., 38G6, 38C8) that could potentially be used as human therapeutic antibodies. We also developed a fluorescently-labeled hepcidin analog (RhoG-hepcidin) by systematically replacing all the non-cysteine amino acids and testing analogs for functionality comparable to unlabeled wild type (WT) hepcidin. Using RhoG-hepcidin and mouse anti-human FPN antibodies, we developed complementary assays that allowed us to investigate the binding of hepcidin to FPN, and to identify molecules that would inhibit the interaction between FPN and hepcidin. Ferroportin-hepcidin antagonists would be expected to mobilize iron by preserving FPN activity in the presence of hepcidin. In the absence of hepcidin, these antagonists would not be expected to affect FPN activity.

Although the monoclonal antibodies were highly specific for FPN, they did not directly antagonize hepcidin binding, as they bound to an extracellular loop of FPN distal from the proposed hepcidin binding site (Taniguchi et al., 2015). The protective effect afforded by these antibodies was reduced at higher hepcidin concentrations, leading us to surmise that they sterically hindered FPN-hepcidin binding with relatively low efficiency. However, an anti-FPN antibody developed by Eli Lilly and Company has been described as binding to the same region (fifth extracellular loop) as our antibodies (Leung et al., 2014), and is currently in a Phase 1 clinical trial. LY2928057, a humanized IgG4 monoclonal antibody that was reported to bind to FPN with high affinity, blocks hepcidin binding to FPN and inhibits hepcidin activity *in vivo*, as measured by increased serum iron levels in cynomolgus monkeys (Leung et al., 2013). A recent report indicated the antibody was well tolerated in patients with CKD with positive changes in iron pharmacodynamics markers; however administration as a single agent did not result in hemoglobin values that met the pre-defined success criterion (Barrington et al., 2016). It is possible that combination with recombinant erythropoietin would lead to larger increases in hemoglobin values as it is possible that iron mobilization alone is not sufficient to enable productive erythropoiesis. To our knowledge, there has only been one other anti-human FPN monoclonal antibody described in the literature; this antibody was used as a tool for cell-based assay, rather than as a potential antagonist (Wallace et al., 2017).

Our reagents and the associated assays also enabled a small molecule screen that identified antagonists of hepcidin-mediated FPN internalization. The assays allowed us to distinguish between different modes of action, i.e., molecules that acted by inhibiting hepcidin binding and those that did not inhibit hepcidin binding, but otherwise inhibited FPN internalization. Of those compounds that inhibited hepcidin binding, the sulfonyl quinoxaline compound **1** was demonstrated to be thiol-reactive and capable of irreversibly binding to cell-expressed FPN. Based on the rate of reaction of compound **1** with cell-expressed, folded FPN vs. a peptide containing a free thiol, we inferred that the compound had some affinity for folded FPN. Recently, another thiol-reactive compound, the vitamin B1-derivative fursultiamine, was identified in a cell-based small molecule screen conducted using cells expressing FPN-GFP; the molecule was shown to inhibit hepcidin binding to FPN *in vitro*, but had a short half-life *in vivo* (Fung et al., 2013).

We previously used these reagents and assays to probe the mechanism of hepcidin-mediated FPN internalization. In companion publications, we, in collaboration with Qiao et al., reported that FPN internalization involved ubiquitination on FPN lysine residues rather than phosphorylation on tyrosine residues (Qiao et al., 2012; Ross et al., 2012). FPN internalization and degradation were formerly understood to require JAK2 activation, phosphorylation of two adjacent FPN tyrosines and STAT3-mediated transcription (De Domenico et al., 2007). This mechanism would have allowed therapeutic intervention by JAK2 modulation, but could not be verified. Validated JAK2 inhibitors were shown to be ineffective at modulating FPN internalization, and Y302/Y303 were not required for FPN internalization, while K240/K258 were required (Ross et al., 2012).

Here we continue to explore the characteristics of the FPN-hepcidin interaction. A previous report indicated that mammalian hepcidin did not bind to FPN at low temperatures (De Domenico et al., 2008). This observation was based on hepcidin binding to a 19 amino acid peptide of FPN spanning amino acids 324–343, termed the hepcidin-binding domain (HBD). The temperature dependency of this interaction was reported to be associated with structural changes in the N-terminal region of hepcidin. Our data showing that RhoG-hepcidin bound to cell surface FPN at 0°C disagrees with this previous report. In addition, detailed structural studies showed that hepcidin's conformation was maintained within the −20 to +53°C range (Jordan et al., 2009).

In addition to showing that hepcidin bound FPN at 0°C, we also found that the interaction is reversible as the RhoG-hepcidin could be displaced by excess unlabeled hepcidin. This is surprising given the current model where hepcidin occupies the central cavity of FPN (Figure 1) and previous reports suggesting that ferroportin-hepcidin interaction may involve, at least transiently, a disulfide exchange (Fernandes et al., 2009; Preza et al., 2011). A model of FPN structure has been proposed based on the structure of a bacterial FPN homolog, BbFPN (Taniguchi et al., 2015). BbFPN contains 12 TM domains forming an N and C lobe which can either assume an “inward facing” or “outward-facing” conformation. By transitioning through these two different conformations, the authors propose a mechanism by which iron could be transported across the cell membrane. The hepcidin binding site can be estimated based on the location of residues shown previously to be important for the hepcidin-FPN interaction. In this model, hepcidin binds in the central cavity between the N and C lobe in the “outward-facing” conformation. Assuming this model is correct, it is unclear how excess unlabeled hepcidin would displace RhoG-hepcidin from this cavity. Given the temperature of our experiments, it is possible that the FPN structure was not fully competent for hepcidin binding and as a result we observed binding to an intermediate conformation between the “outward facing” or “inward-facing” conformations. In this scenario, RhoG-hepcidin was loosely bound with insufficient FPN residue contact in the central cavity to form a stable interaction.

Differences in FPN glycosylation and cellular localization were reported in mouse duodenal vs. bone marrow-derived macrophages, suggesting that glycosylation may be important for FPN localization and function in different tissues (Canonne-Hergaux et al., 2006). We treated FPN-expressing cells with tunicamycin to inhibit N-linked glycosylation, and subsequently treated these cells with hepcidin to determine if FPN glycosylation was required for hepcidin-mediated internalization. We found glycosylation was not necessary for hepcidin binding or FPN degradation, as (1) tunicamycin-treated cells internalized RhoG-hepcidin comparably to untreated cells and (2) non-glycosylated FPN was completely degraded after treatment with hepcidin. Interestingly, we discovered that one of our antibodies, Ab 38C8, did not recognize FPN after treatment of cells with tunicamycin or when the protein was enzymatically deglycosylated. There is one extracellular N-linked glycosylation site (N434), which is in the same EC loop to which most of our antibodies bind. Ab 38C8 is an antibody for which we were unable to resolve a linear epitope, and these results suggest that the antibody recognizes a region containing glycosylated N434.

In summary, we developed specific reagents for interrogating the FPN-hepcidin interaction. Our monoclonal antibodies are sensitive, specific for FPN and useful for different applications, including immunoblotting, immunofluorescence, flow cytometry and immunohistochemistry. These antibodies, along with RhoG-hepcidin, provided tools to visually follow hepcidin-mediated internalization and to identify novel molecules that inhibit interaction with FPN. In these investigations involving multiple modalities including antibodies, peptides and small molecules, we discovered useful tools that advanced the understanding of the FPN-hepcidin interaction.

## Author contributions

SR and TA conceived and designed experiments, analyzed and interpreted data and drafted the manuscript. LM, JL, KB, JA, and AW designed and synthesized molecules, interpreted data and critically revised the manuscript. JR interpreted data and critically revised the manuscript. All authors approved the final version of the manuscript and agree to be accountable for all aspects of the work.

### Conflict of interest statement

The authors declare that the research was conducted in the absence of any commercial or financial relationships that could be construed as a potential conflict of interest.
