# Supplementary material for: Identification of Antibody and Small Molecule Antagonists of Ferroportin-Hepcidin Interaction
Source: Front Pharmacol. 2017 Nov 21;8:838. doi: 10.3389/fphar.2017.00838 (PMC5702341; doi:10.3389/fphar.2017.00838)
Supplement: Supplementary file 1 [file Presentation1.PDF]

Supplementary Material

Identification of Antibody and Small Molecule Antagonists of Ferroportin-Hepcidin Interaction

Sandra Ross, Kaustav Biswas, James Rottman, Jennifer Allen, Jason Long, Les P. Miranda, Aaron Winters, Tara Arvedson*

*** Correspondence: Tara Arvedson: taraa@amgen.com**

Supplementary Figures

Supplementary Figure 1

A

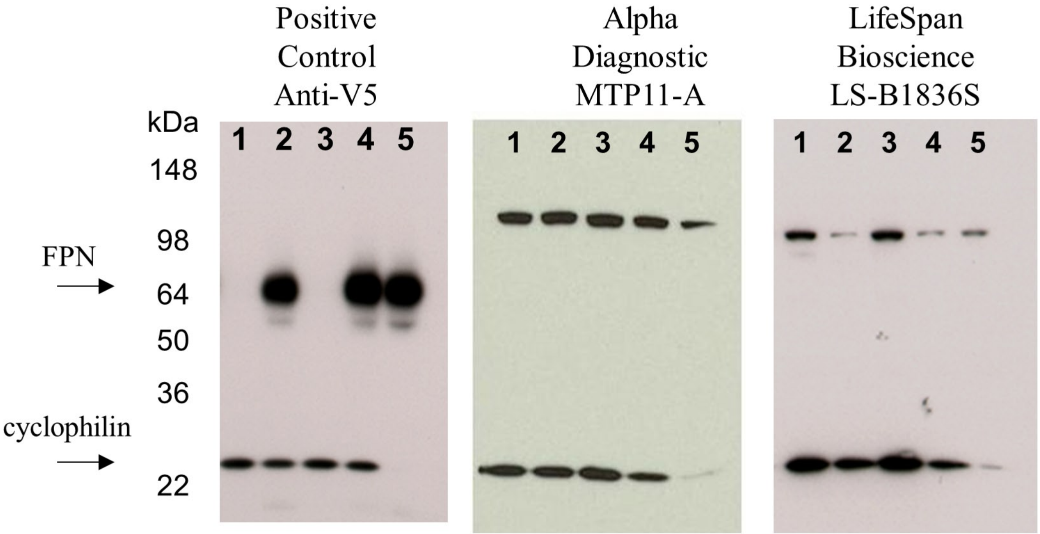

B

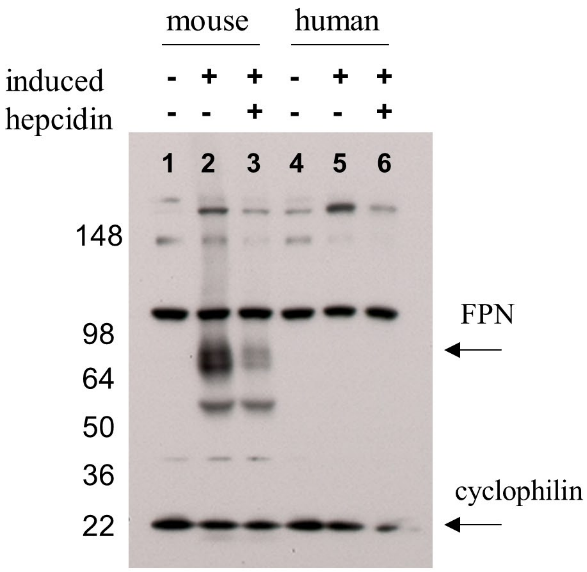

Supplementary Figure 1. Two commercially-available anti-FPN antibodies did not detect human FPN. **(A)** T-REx™/Fpn-V5 cells were treated +/- 80nM siRNA reagents for 48 hours and lysed. Lane 1, uninduced cells; lane 2, induced cells; lane 3, induced cells + FPN siRNA; lane 4, induced cells + non-silencing siRNA control; lane 5, induced cells + cyclophilin siRNA. Blots were probed with vendor-recommended as listed in Materials and Methods. **(B)** Uninduced and induced cells expressing mouse (T-REx™/mu Fpn-V5/BLA, lanes 1-3) or human (T-REx™/Fpn-V5, lanes 4-6) FPN were treated (-/+) with 500nM hepcidin for 18 hours; 20 µg lysate/lane was loaded and the blot was probed with 5 µg/ml Alpha Diagnostics MTP11-A antibody.

Supplementary Figure 2

```
HUMAN       MTRAGDHNRQRGCCGSLADYLTSAKFLLYLGHSLSTWGDRMWHFAVSVFLVELYGNSLLL     60
MOUSE       MTKARDQTHQEGCCGSLANYLTSAKFLLYLGHSLSTWGDRMWHFAVSVFLVELYGNSLLL     60
RAT         MTKSRDQTHQEGCCGSLANYLTSAKFLLYLGHSLSTWGDRMWHFAVSVFLVELYGNGLLL     60
            **:: *:.:*.*******:************************************.***

HUMAN       TAVYGLVVAGSVLVLGAIIGDWVDKNARLKVAQTSLVVQNVSVILCGIILMMVFLHKHEL     120
MOUSE       TAVYGLVVAGSVLVLGAIIGDWVDKNARLKVAQTSLVVQNVSVILCGIILMMVFLHKNEL     120
RAT         TAVYGLVVAGSVLVLGAIIGDWVDKNARLKVAQTSLVVQNVSVILCGIILMMVFLHKNEL     120
            *********************************************************:**

HUMAN       LTMYHGWVLTSCYILIITIANIANLASTATAITIQRDWIVVVAGEDRSKLANMNATIRRI     180
MOUSE       LTMYHGWVLTVCYILIITIANIANLASTATAITIQRDWIVVVAGENRSRLADMNATIRRI     180
RAT         LNMYHGWVLTVCYILIITIANIANLASTATAITIQRDWIVVVAGENRSRLADMNATIRRI     180
            *.******** **********************************:**:**:********

HUMAN       DQLTNILAPMAVGQIMTFGSPVIGCGFISGWNLVSMCVEYVLLWKVYQKTPALAVKAGLK     240
MOUSE       DQLTNILAPMAVGQIMTFGSPVIGCGFISGWNLVSMCVEYFLLWKVYQKTPALAVKAALK     240
RAT         DQLTNILAPMAVGQIMTFGSPVIGCGFISGWNLVSMCVEYFLLWKVYQKTPALAVKAALK     240
            ****************************************.****************.**

HUMAN       EEETELKQLNLHKDTEPKPLEGTHLMGVKDSNIHELEHEQEPTCASQMAEPFRTFRDGWV     300
MOUSE       VEESELKQLTSPKDTEPKPLEGTHLMGEKDSNIRELECEQEPTCASQMAEPFRTFRDGWV     300
RAT         VEESELKQLTSPKDTEPKPLEGTHLMGEKDSNIRELECEQEPTCASQIAEPFRTFRDGWV     300
             **:*****.  *************** *****:*** *********:************

HUMAN       SYYNQPVFLAGMGLAFLYMTVLGFDCITTGYAYTQGLSGSILSILMGASAITGIMGTVAF     360
MOUSE       SYYNQPVFLAGMGLAFLYMTVLGFDCITTGYAYTQGLSGSILSILMGASAITGIMGTVAF     360
RAT         SYYNQPVFLAGMGLAFLYMTVLGFDCITTGYAYTQGLSGSILSVLMGASAITGIMGTVAF     360
            *******************************************:****************

HUMAN       TWLRRKCGLVRTGLISGLAQLSCLILCVISVFMPGSPLDLSVSPFEDIRSRFIQGESITP     420
MOUSE       TWLRRKCGLVRTGLFSGLAQLSCLILCVISVFMPGSPLDLSVSPFEDIRSRFVNVEPVSP     420
RAT         TWLRRKCGLVRTGLFSGLAQLSCLILCVISVFMPGSPLDLSVSPFEDIRSRFIHEEAVSS     420
            **************:*************************************:: * ::

HUMAN       TK-IPEI--TTEIYMSNGSNSANIVPETSPESVPIISVSLLFAGVIAARIGLWSFDLTVT     477
MOUSE       TTKIPETVFTTEMHMSNMS----NVHEMSTKPIPIVSVSLLFAGVIAARIGLWSFDLTVT     476
RAT         TTKIPETEMLMSNVSNVVN----TVHEMSTKSVPIISVSLLFAGVIAARIGLWSFDLTVT     476
            *. ***     .   .  .     * * * : :**:************************

HUMAN       QLLQENVIESERGIINGVQNSMNYLLDLLHFIMVILAPNPEAFGLLVLISVSFVAMGHIM     537
MOUSE       QLLQENVIESERGIINGVQNSMNYLLDLLHFIMVILAPNPEAFGLLVLISVSFVAMGHLM     536
RAT         QLLQENVIESERGIINGVQNSMNYLLDLLHFIMVILAPNPEAFGLLVLISVSFVAMGHLM     536
            **********************************************************:*

HUMAN       YFRFAQNTLGNKLFACGPDAKEVRKENQANTSVV      571
MOUSE       YFRFAQKTLGNQIFVCGPDEKEVTDENQPNTSVV      570
RAT         YFRFAQKTLGNQIFVCAPDEKEVTDESQPNTSVV      570
            ******:****::*.*.** *** .*.* *****
```

Supplementary Figure 2. Monoclonal antibodies bind to the extracellular loop between transmembrane helices 9 and 10 (yellow highlight), a region that is not well conserved. Cys326 (green highlight), essential for hepcidin binding, is distal to the antibody-binding region and is predicted to be within the central cavity (Taniguchi *et al.* 2015).

Supplementary Figure 3

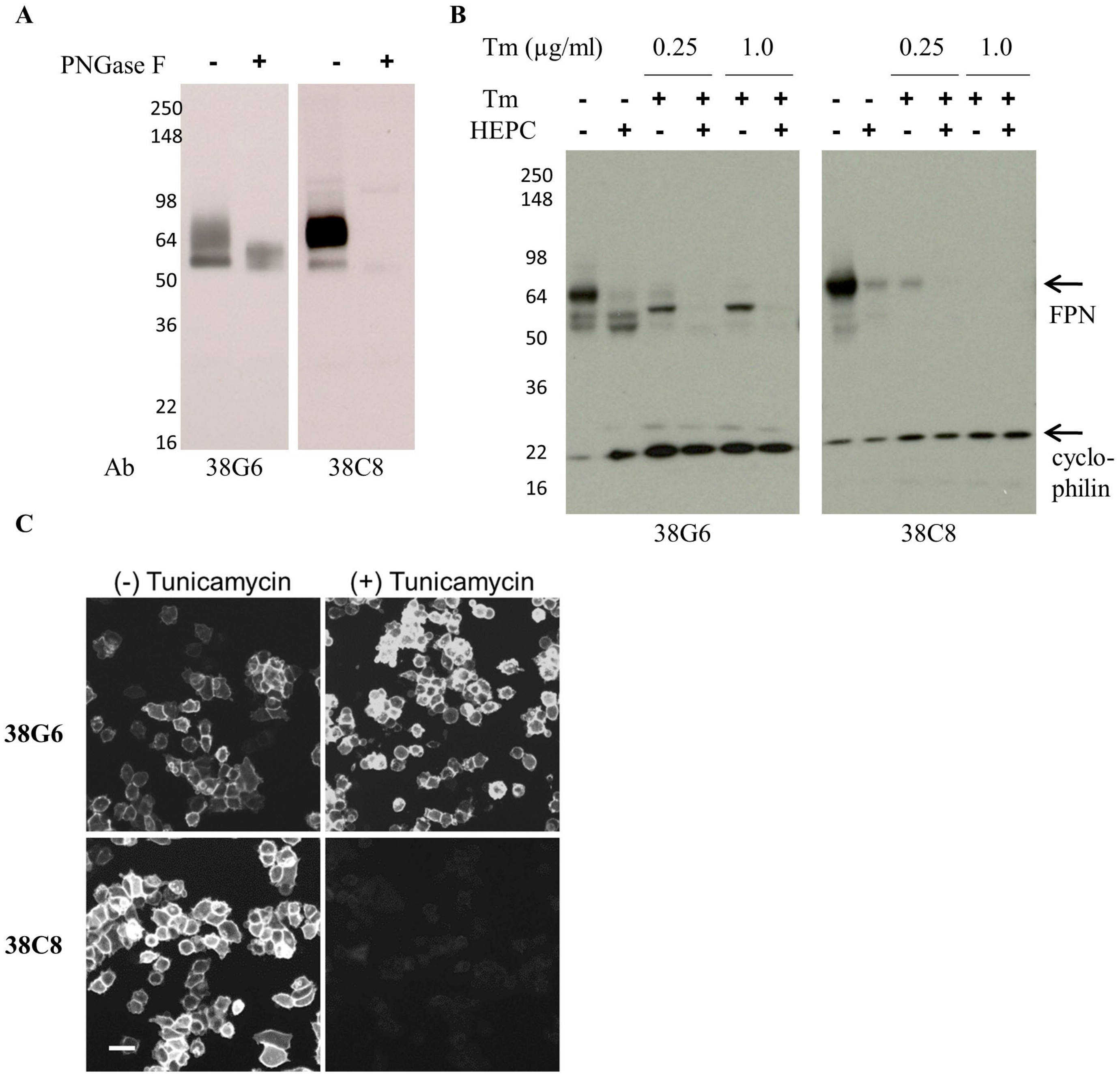

Supplementary Figure 3. Anti-FPN antibody 38C8 does not recognize unglyclosylated FPN. **(A)** Induced T-REx™/Fpn-V5 cell lysates were immunoprecipated with anti-V5 antibody prior to treating with -/+ PNGase F for 3 hours; blots were probed with1 µg/ml 38G6 (left panel) or 20 µg/ml 38C8 (right panel). **(B)**, Induced T-REx™/Fpn-V5 cells were treated -/+ 0.25 or 1 µg/ml tunicamycin (Tm) for 18 hours followed by treatment with -/+ 500nM hepcidin (HEPC) for 5 hours prior to making lysates. FPN was detected with 1 µg/m 38G6 (left panel) or 0.5 µg/ml 38C8 (right panel); cyclophilin (22 kDa) was used as a loading control. **(C)** Induced T-REx™/Fpn-V5 cells were treated for 18 hours with 0.25µg/ml tunicamycin; FPN was detected with 2 µg/ml 38G6 (top panels) or 38C8 (bottom panels) after fixing; scale bar = 20 µm.
